# A comprehensive phylogeny of mammalian *PRNP* gene reveals no influence of prion misfolding propensity on the evolution of this gene

**DOI:** 10.1371/journal.ppat.1013257

**Published:** 2025-06-25

**Authors:** Cristina Sampedro-Torres-Quevedo, Hasier Eraña, Jorge M. Charco, Carlos M. Díaz-Domínguez, Maitena San-Juan-Ansoleaga, Eva Fernández-Muñoz, Nuno Gonçalves-Anjo, Josu Galarza-Ahumada, Ana R. Cortazar, Roberto F. Nespolo, Julian F. Quintero-Galvis, Africa Manero-Azua, Diego Polanco-Alonso, Adrián Gaite-Reguero, Íñigo Olalde, Urko M. Marigorta, Guiomar Perez de Nanclares, Ana M. Aransay, Joaquín Castilla

**Affiliations:** 1 Center for Cooperative Research in Biosciences (CIC BioGUNE), Prion Lab and Integrative Genomics Lab, Basque Research and Technology Alliance (BRTA), Derio, Spain; 2 Centro de Investigación Biomédica en Red de Enfermedades infecciosas (CIBERINFEC), Carlos III National Health Institute, Madrid, Spain; 3 ATLAS Molecular Pharma S. L., Derio, Spain; 4 Center for Cooperative Research in Biosciences (CIC BioGUNE), Genome Analysis Platform, Basque Research and Technology Alliance (BRTA), Derio, Spain; 5 Instituto de Ciencias Ambientales y Evolutivas, Universidad Austral de Chile, Valdivia, Chile; 6 Center of Applied Ecology and Sustainability (CAPES), Facultad de Ciencias Biológicas, Universidad Católica de Chile, Santiago, Chile; 7 Millenium Institute for Integrative Biology (iBio), Santiago, Chile; 8 Departamento de Ciencias, Facultad de Artes Liberales, Universidad Adolfo Ibáñez, Santiago, Chile; 9 Molecular (Epi)Genetics Laboratory, Bioaraba Health Research Institute, Araba University Hospital, Vitoria-Gasteiz, Spain; 10 BIOMICs Research Group, Department of Zoology and Animal Cell Biology, University of the Basque Country UPV/EHU, Vitoria-Gasteiz, Spain; 11 IKERBASQUE, Basque Foundation for Science, Bilbao, Spain; 12 Centro de Investigación Biomédica en Red de Enfermedades Hepáticas y Digestivas (CIBERehd), Madrid, Spain; National Institutes of Health, NIAID, UNITED STATES OF AMERICA

## Abstract

Prion diseases are invariably fatal neurodegenerative diseases that affect some mammalian species, including humans. These diseases are caused by the misfolding of the cellular prion protein (PrP^C^) into a pathologic isoform (PrP^Sc^). The prion protein is highly conserved across mammals. However, some species present lower susceptibility to prion diseases than others. This behavior is likely explained by the resistance of these animal species’ prion proteins to acquire a pathological conformation. Therefore, the tertiary structure and interspecific variations encoded in the primary structure determine a PrP proneness to misfolding. For this reason, we studied the *PRNP* gene from a phylogenetic perspective, potentially unveiling evolutionary events related to prion diseases. We generated a database of mammalian *PRNP* sequences and constructed phylogenetic trees based on nucleotide sequence variations. We aligned 1146 *PRNP* gene sequences from 901 different mammalian species and built a *PRNP* gene-based phylogenetic tree. Classical phylogenetic orders tend to maintain their clustering in the *PRNP* gene tree. Nonetheless, the few differences found may shed some light on potential evolutionary constraints posed by prion disorders. Moreover, this phylogenetic study was combined with an *in vitro* misfolding study. Protein Misfolding Shaking Amplification (PMSA) was used to evaluate the tendency of many of these proteins to misfold. This comprehensive analysis spanned a wide range of mammalian prion protein sequences and included analysis of different variants with a focus on the human rs1799990 locus (c.385A > G, p.Met129Val). This variant, widely linked to prion disease susceptibility in humans, is explored in the context of its evolutionary origins. All in all, our *PRNP* gene-based tree, despite showing some topological differences with the reference species tree that could be in some cases related to prion disease susceptibility, is not significantly distinct. Indicating that the proneness of a PrP variant to misfold spontaneously has not shaped the evolution of this gene.

## Author summary

Prion diseases are rare and complex neurodegenerative disorders that affect humans, and other mammals, such as cattle or sheep. They are caused by a poorly understood conformational or structural change of normal, physiological prion protein. This abnormally structured form, known as prion, acquires the capacity to induce the same transformation to surrounding physiological prion proteins, leading to disease. Although this protein is very similar in all mammals, prion diseases have only been described in some species. For this reason, we decided to look at as many mammal prion protein sequences as possible and analyze the evolution of this protein in this group of species, to compare it with the tendency of these species’ prion proteins to adopt a toxic conformation. Our results indicate that prion diseases have not shaped the evolution of the prion protein in mammals. Our study provides an insight into the molecular evolution of prion proteins. For example, it has allowed us to look at the evolution of the human prion protein and compare it to older humans and Neanderthals, which has identified a change that is unique to the human species. Thus, although some evolutionary differences could be related to the differential susceptibility of the prion protein variant to misfold, our results indicate that prion diseases have not significantly influenced the evolution of the gene that encodes the prion protein.

## Introduction

Transmissible spongiform encephalopathies (TSEs), also known as prion diseases, are invariably fatal, transmissible, and rapidly progressing neurodegenerative disorders affecting humans and several mammals. The causal agent behind these diseases is the cellular prion protein (PrP^C^), which undergoes a misfolding process turning into an aggregation-prone, conformer known as PrP^Sc^. While the cellular protein is soluble, rich in α-helix domains, and protease-sensitive, the pathology-associated isoform is insoluble, rich in β-sheet domains, partially protease-resistant, and, most importantly, able to induce its aberrant conformation to the cellular counterpart, forming aggregates. This ability to induce the aberrant conformation to the PrP^C^ allows the disease to propagate in the central nervous system of affected individuals, leading to neurodegeneration through yet unknown pathways, and is central to the transmissibility among individuals [1].

Prion diseases can be classified depending on their etiology. The acquired forms occur through ingestion or introduction, through iatrogenic means, of exogenous PrP^Sc^. In familial or hereditary forms of TSE, the disease develops due to the existence of pathogenic variants in the gene encoding the PrP^C^ protein (*PRNP*), which putatively increase the protein’s propensity to misfold into PrP^Sc^ [2]. Once generated, this PrP^Sc^ initiates the auto-propagative process mentioned in the preceding paragraph, leading to the development of genetic prionopathy. In the sporadic forms of the disease, the PrP^Sc^ appears purportedly due to a low-frequency event of spontaneous misfolding of the wild-type PrP^C^. This latter type is, in fact, the most common form of prion disease in humans, accounting for 85–90% of cases. In contrast, genetic forms account for around 10–15% of cases, and acquired forms have almost been eradicated in humans [3]. Although almost non-existent at present, the acquired types of prion disease were of significant importance for human health during the mad cow disease crisis, when a prion disease (vCJD, variant Creutzfeldt-Jakob disease) arose from consumption of infectious meat from cows suffering from bovine spongiform encephalopathy (BSE). Humans have coexisted with scrapie, a prion disease affecting sheep and goats, for centuries without reports of zoonosis, making the mad cow disease crisis an unprecedented event that raised awareness among authorities about the risk posed by the interspecies transmissibility of prions. This epidemic and zoonotic event, as well as reports of experimental inoculations, illustrate the capacity of prions for interspecies transmission and the fact that some prion diseases, but not others, can infect species different from those in which they originated, a phenomenon determined by the so-called interspecies or transmission barrier [4]. In cases in which a strong transmission barrier exists, incomplete attack rates and prolonged incubation periods are typically observed upon inoculation of the new host, whereas for weak or non-existent barriers, 100% attack rates and incubation periods similar to those in the original host are observed.

Crucial to the ability to propagate at the expense of a given PrP^C^ substrate is the concept of prion strains. Prion strains are different prion isolates with distinct biological and biochemical properties, varying for example in incubation times, histopathological lesion profiles, clinical signs, and relative proteinase resistance, among others [5]. These differences are thought to be encoded in the tertiary structure of the protein, and it is the ability of a given PrP^C^ protein to adopt the distinct conformation of an infectious PrP^Sc^ what determines strain compatibility and thus, the presence or absence of an interspecies barrier. This ultimately depends on the primary structure of the protein, as illustrated by experiments in *PRNP* knockout mice expressing a hamster-mouse chimeric *PRNP* protein, which show differential susceptibility to distinct prion strains [6]. Moreover, two genotypic mouse variants: *Prnp*^*A*^ and *Prnp*^*B*^, have been described, which present different incubation times when inoculated with the same prion inoculum [7]. All these results point to the underlying amino acid sequence as the key regulator of prion disease susceptibility. However, prediction of transmission barriers based solely on *PRNP* sequence is not yet possible, as they are conformational diseases.

In this regard, it is important to consider whether prion diseases could potentially affect all animal species with a *PRNP* gene, or if there is a limited host range for prion propagation. Understanding this could help reduce the risk of new prion disease outbreaks, interspecies transmission events among cohabiting animals, or even zoonosis. In fact, in addition to the relatively few naturally occurring prion diseases that have been described in humans and other mammalian species, several prion diseases have been induced under laboratory conditions. This includes not only experimental inoculation of naturally occurring prion diseases [8–10] but also the generation of novel prions through *in vitro* prion propagation systems [11–21]. These forms of prion diseases have been described mostly in rodent species for which no naturally occurring prion disease had been reported, highlighting the fact that the susceptibility of mammalian species to TSEs may be substantially higher than previously described.

Although the *PRNP* gene is frequently described as highly conserved across mammals, direct quantification of this conservation has often been limited. Comparative analyses offer stronger support: a dN/dS ratio of 0.0105 has been reported for the bovine *PRNP* gene [22], placing it among the most conserved genes, comparable to histones and ubiquitin. Consistently, our analysis of mammalian *PRNP* sequences revealed an average dN/dS ratio of 0.18, indicative of strong purifying selection acting across diverse species. This could explain the occurrence of prion diseases across different families and their ability to be transmitted across different species. Furthermore, homologues have been identified in several classes of vertebrates such as avian [23,24], reptile [25], amphibian [26], and fish species [27]. All of these show conservation of protein features such as the signal peptide, repetitive region, hydrophobic region, glycosylation sites near a disulfide bridge, and a GPI anchor site. Nevertheless, to date, prion diseases have only been reported in mammalian species. This could be due to differences in the N-terminal repeated sequences or C-terminal specific sequences, which vary within mammals and other vertebrate groups [27]. Regardless of the underlying explanation, this suggests that there may be a limited number of PrP^C^ sequences that can adopt the conformation that grants prions their neurotoxic and self-propagating features.

However, what determines the susceptibility of any given PrP^C^ sequence to acquire a prion conformation remains completely unknown. In fact, due to the outstanding conservation found among mammalian *PRNP* sequences, it is quite challenging to ascertain the molecular determinants that drive the differences in prion disease susceptibility among mammalian species. Notably, extremely similar sequences have shown substantial differences in terms of capacity to misfold and give rise to a prionopathy. For instance, rabbits were considered to be resistant to prion diseases for a long time, until *in vitro* experiments showed that, although with difficulties, it was possible to misfold the rabbit PrP^C^. Subsequently, both rabbits and transgenic mice expressing rabbit PrP were infected with several prion inocula [28,29]. The low susceptibility of rabbit to prion infection, despite their PrP being very similar to that of highly susceptible animals such as mice and other rodent species, highlights that although sequence similarity plays a role, sometimes the presence of certain residues in a given amino acidic context can be more important than overall sequence similarity [30]. Another example is provided by the order Carnivora. Among this order, canine species have previously been shown to present resistance against prion diseases when compared to highly similar species in the same order, which are susceptible to prionopathies [31–33]. This resistance was hypothesized to be due to the presence of an acid residue (Asp or Glu) at codon 163, in lieu of the more common asparagine (Asn). This modification is not present in other more susceptible members of the Carnivora order, despite having high sequence similarity. It has been suggested that this variant could have appeared as a result of natural selection in canids, which feed on carrion [34]. Moreover, it underscores that there must be a more complex mechanism involving the donor strain and receptor PrP^C^ and the capability of one to accommodate the other and adopt its conformation. Should this be the case, it is possible that no such thing as a species resistant to prion disease propagation exists, just a lack of known strains that are able to infect certain species [35,36].

It is worth noting that molecular evolution is normally governed by the physiological function of a protein, where mutations that impede its function are avoided and those that enhance its capacity to carry out such function might be passed on [37]. However, since the function of the PrP protein remains evasive, and lack of function mutants do not seem to have major complications but rather subtle effects at advanced ages [38], we wondered if the driver of evolution for this protein could had been their susceptibility to prion diseases rather than the function of the physiological isoform of the protein, as the only known dramatic effect of alterations in the *PRNP* gene are related to prion disease susceptibility [39].

Thus, in the light of the direct relationship between PrP amino acid sequence and susceptibility to prion disease, the aim of this paper is to increase the knowledge on mammalian *PRNP* sequences and to delve into their variability and the potential effects of TSEs on *PRNP* evolution. Here we aim to evaluate if the presence of prionopathies has shaped the evolution of the *PRNP* gene.

To this end, we have gathered the most comprehensive collection of mammalian *PRNP* sequences, including in our analysis all available annotated sequences from public databases, sequences extracted and annotated from whole genome sequencing projects, and new ones obtained from tissue and body fluid samples from zoo and shelter specimens. A total of 1150 *PRNP* sequences were analyzed using the Bayesian Evolutionary by Sampling Trees package (BEAST) to build a *PRNP*-based phylogenetic tree. By comparing the arrangement of mammalian families in this tree to the latest timetree describing mammalian evolution [40], we expect to uncover PrP-driven differences that might be related to prion disease susceptibility. For this, in addition to the information available in the literature about the relative susceptibility of distinct mammalian species, we have developed a new *in vitro* method, Protein Misfolding Shaking Amplification (PMSA), which allows spontaneous recombinant PrP misfolding into *bona fide* prions, and thus enables evaluation of the misfolding proneness of PrP variants or motifs of interest [41]. Ultimately, our efforts will give rise to the most complete PrP-based phylogenetic tree for the class Mammalia, offering insight into unique motifs or PrP variants that could be related to prion disease susceptibility and providing relevant information about the limits imposed by nature in terms of PrP variants that are able to acquire an infectious conformation.

## Results and discussion

### Comprehensive collection and analysis of mammalian *PRNP* sequences

Our extensive efforts to gather *PRNP* sequences resulted in a collection of 1146 sequences from 901different mammalian species, representing the most comprehensive dataset to date. This collection encompasses sequences from 28 out of the 29 existing mammalian orders (note that the latest classifications group Cetacea (whales, dolphins, and porpoises) and Artiodactyla (Even-toed ungulates, such as pigs, deer, cows, camels, and giraffes) orders under the Cetartiodactyla order, making the total number of mammalian orders 28, but for this study they have been considered separately), with only the Paucituberculata order (a group of marsupial South American opossums with only 7 extant species) remaining unrepresented (S2 Table).

Focusing on the core region from codon 23–231, which represents the mature protein subject to misfolding and potential evolutionary pressure, we aligned and merged identical sequences. This refinement process resulted in 1025 unique sequences (S2 Table), providing a robust foundation for our phylogenetic analysis. The manual curation process, performed using MEGAX software, revealed no hypervariable regions that required removal. Instead, the primary differences addressed were algorithmic oversights in aligning certain features. Particular attention was given to the octapeptide repeat (OR) area and the C-terminal region of the protein, which exhibited the highest variability. This curated alignment was further refined by generating consensus sequences to minimize the effect of low bootstrap nodes. The resulting alignment of 357 sequences (S1 Text) forms the basis for our subsequent phylogenetic analysis and investigation into potential evolutionary pressures related to prion disease susceptibility across mammalian species.

### Assembling the mammalian *PRNP* phylogenetic tree

Using the curated alignment of mammalian *PRNP* sequences, we generated a phylogenetic tree to explore potential relationships between *PRNP* sequence diversity and misfolding propensity, considering Monotremata (egg-laying mammals including the platypus and echidnas) as the root or outgroup. The tree structure reflects both the similarities between sequences (topology) and the evolutionary change (branch lengths) [42].

**Fig 1:**
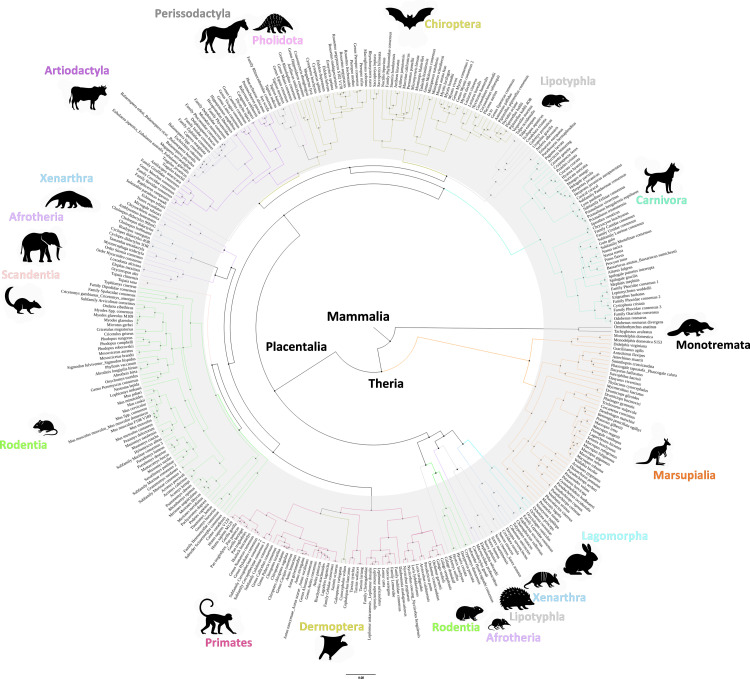
Prion protein nucleotide sequences based phylogenetic tree compiled using BEAST software. 1146 different nucleotide sequences from 901 different mammalian species were used to perform the analysis as described. Coloured clusters represent different mammalian orders, consistent with the latest species trees. The mammalian orders tend to maintain their clusterization in the *PRNP* gene tree, albeit with some exceptions such as the clusterization of most Afrotheria (A major clade of mammals with African origins, including elephants, manatees, aardvarks, and tenrecs) with Primates and Dermoptera (order of colugos or “flying lemurs,” gliding mammals from Southeast Asia), as well as the nesting of Lipotyphla (an obsolete order formerly used to group insectivorous mammals such as hedgehogs, shrews, and moles, now largely replaced by Eulipotyphla) within the Chiroptera (bats) species.

The final tree (Fig 1) was generated after addressing branches with low bootstrap support (<50%) through the creation of consensus sequences where appropriate. This refined tree provides a more robust representation of *PRNP* sequence relationships across mammalian species. The tree with the bootstrap values is represented in S1 Fig.

**Fig 2:**
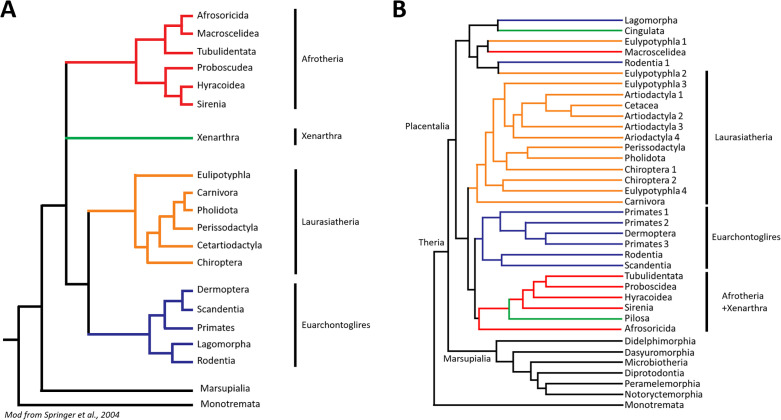
A standard phylogenetic tree of species **(A) compared to the tree obtained in this study using the PRNP gene (B). Both trees illustrate orders or higher organisational clades whenever possible.** The tree on the left (A) represents a classical mammalian evolution tree (modified from Springer 2004 [43]), whereas the tree on the right (B) displays the results obtained in this study. Classifications above the order level are represented to the right of the tree, while preceding classifications are shown at the corresponding nodes. In the tree obtained in this study (B) the colours correspond to those used in the example tree (A) for the same higher clades, facilitating comparison between the two. While species belonging to the same order tend to be clustered together in the *PRNP* gene tree, higher classifications show less conservation. However, the trees exhibit substantial similarity, and the few differences presented could offer insight into *PRNP* evolution.

Analysis of the resulting tree reveals a topology that is generally consistent with current mammalian taxonomy. Most species from the same order are supported as clades, although exceptions to perfect conservation exist (Fig 2) [43]. This alignment between the *PRNP*-based phylogeny and established taxonomic classifications suggests that *PRNP* evolution largely reflects overall mammalian evolution. Nevertheless, we have identified several noteworthy mismatches. These discrepancies may indicate the common occurrence of specific motifs and residues potentially relevant to protein misfolding. Consequently, in the subsequent sections, we will assess the misfolding propensity of these diverse PrP proteins *in vitro*. Our aim is to test the hypothesis that discrepancies with the overall mammalian evolutionary tree could reveal motifs that may play a significant role in the misfolding process.

### Phylogenetic analysis of mammalian *PRNP* sequences: insights and divergences from classical taxonomy

The relationship between living species has been a subject of scientific inquiry for centuries. Phylogeny, as a field, is as dynamic as the species it investigates, undergoing constant revision, scrutiny, and change. Species classifications are frequently updated, with organisms being regrouped into different genera or families, or reclassified entirely. A notable example is the reassessment of the order Insectivora, which revealed that its constituent species actually belong to several distinct orders [44]. While major changes to the grand scheme of orders are infrequent, modifications within orders are more common and can even affect species nomenclature. As a result, some species have undergone name changes between the outset of this study and the uploading of their sequences to GenBank. These taxonomic challenges and discrepancies arise from incomplete data on species evolution and the inconsistent incorporation of fossil records to phylogenetic studies [45]. Rapid speciation and diversification events pose particular challenges for existing algorithms in accurately predicting evolutionary relationships [46]. This complexity is further evident in the distinction between gene trees and species trees. Gene trees are derived from single-gene phylogenies and reflect the evolutionary pathway of individual genes, while species trees, based on multiple genes, represent the broader evolutionary trajectories of species [47]. Despite their differences, gene trees remain valuable tools in evolutionary biology. Significant discrepancies between gene and species trees may indicate selective pressure on that particular gene, potentially suggesting adaptive responses [48]. Nevertheless, the aim of this paper is not to infer phylogenetic relations from a gene tree but to evaluate the potential impact of prion diseases on mammalian *PRNP* evolution. Therefore, we hypothesized that if prion diseases susceptibility could have influenced *PRNP* evolution, we would find significant differences between our *PRNP* gene-based phylogenetic tree and the species tree from mammals, maybe with prion resistance motifs enriched in groups that could have been exposed to selective pressure posed by prion infection. For that purpose, we conducted a comparison between our *PRNP* tree, and a species tree generated *ad hoc* with VertLife [49] using the Robinson-Foulds (RF) distance metric [50]. This analysis showed an RF distance of 0.52 between both trees, indicating that 52% of bipartitions or splits differ between them. A permutation test using 1,000 randomly sampled tree topologies revealed that the observed RF distance fell well within the range of permuted values (p = 1), indicating that the topological divergence between the gene tree and the species tree is not statistically significant, and thus, suggesting no influence of prion diseases in the evolution of this gene among mammals. When visualized side-by-side (S2 Fig), the two trees show some topological differences despite containing the same set of taxa. Nevertheless, most of these differences are within the same cluster and probably explained due to the similarities of the *PRNP* genes and the difficulties to resolve inner nodes. This is supported by an average of 0.13 base differences per site from averaging over all sequence pairs as calculated by MEGAX, with a maximum difference between sequences of 0.3. There are however, three clusters that stand out in this analysis as highly different in the *PRNP* gene tree, one representing the cingulate (armadillos) and some species of Afrotheria (a clade of mostly African mammals which include elephants), which seem to be more similar to other mammals in terms of *PRNP* gene than expected. The second one corresponds to the Lagomorpha (the order encompassing rabbits and hares, among others) which also is very far away from its traditional position grouped with rodents. Lastly, there is a cluster of rodents including squirrels that seem to be further away from the rest of rodents and closer to primates in terms of *PRNP* gene sequence.

In this section, we will provide a detailed analysis of the most relevant aspects observed in the *PRNP*-based phylogenetic tree, drawing comparisons with the most comprehensive taxonomic tree available. This information will be primarily organized and presented through a detailed description of the most significant nodes in the newly generated phylogenetic tree, allowing for a systematic exploration of the key evolutionary relationships and divergences revealed by our *PRNP*-based analysis.

The node Atlantogenata, traditionally proposed as a clade comprising Xenarthra (a basal placental mammal clade including armadillos, anteaters, and sloths) and Afrotheria, was not supported by the *PRNP* phylogeny and effectively disappeared. Instead, its members were distributed across two different clusters. The first cluster included representatives from one out of six Afrotheria members: Macroscelidea (order of elephant shrews or sengis, small insectivorous mammals, endemic to Africa). This first cluster groups the Macroscelidea with the Cingulata (order comprising all armadillos), one of the two orders included in the superorder Xenarthra (Fig 2). However, although this branching supports the idea that these groups’ *PRNP* gene is the most divergent from all mammalian species, they are also grouped with representatives from other orders. Namely, the Lagomorpha, which is no longer clustered with Rodentia and Primates, some Rodentia species belonging to the infraorder Hystricognathi (a suborder including porcupines, guinea pigs, capybaras, and other large-bodied South American rodents), and some representatives from the orders Erinaceomorpha (formerly used order now often merged into Eulipotyphla; includes hedgehogs and gymnures) and Soricomorpha (an outdated grouping formerly including shrews and moles). This clade presents the most divergent *PRNP* genes from all the ones included in the study. This cluster is branched away from the rest of the Boreoeutheria. The second cluster is composed of the order Pilosa (sloths and anteaters) and all of the included representatives from the rest of the Afrotheria orders (Sirenia, Proboscidea, Hyracoidea, Macroscelidea and some Afrosoricida species). This cluster appeared grouped closer to primates and rodents in the *PRNP* tree. This distribution suggests that some Afrotheria *PRNP* sequences are more similar to those of Euarchontoglires (a supraprimate clade including rodents, lagomorphs, primates, tree shrews, and colugos) than to other Afrotheria or more closely related evolutionary groups, representing the most significant deviation from the species phylogenetic tree observed in our *PRNP* analysis. Nevertheless, all Afrotherian species within this clade remain grouped together, indicating their closer relationship to each other than to any other species in this study. The observed discrepancy between the *PRNP* gene tree and classical phylogeny is particularly puzzling given that Afrotheria was one the most challenging clades to characterize morphologically, only being recognized and grouped through various genetic analyses [51], underscoring the importance of distinguishing between gene trees and species trees. Consequently, a single branch in a species tree can encompass multiple branches of a gene tree, with potentially different evolutionary trajectories [52]. Coalescence models demonstrate that gene coalescence can occur before species branching [53]. A similar process might explain the greater similarity of Afrotherian *PRNP* to that of other Euarchontoglires. Nevertheless, this process is more common in trees with short branches since the common ancestor [53]. Given the presumed distant common ancestor of Afrotheria and Euarchontoglires, their similarity could indicate convergent evolution. Convergent evolution at gene level can result from independent mutations in different species or the evolution of an ancestral genetic variant, often influenced by environmental factors that minimize pleiotropic effects while maximiszing adaptation [54]. This raises intriguing questions: what might have driven this potential convergent evolution? Could a PrP more similar to that of Primates confer a beneficial effect to the Afrotheria? While TSEs have been described in several primate species, there is no available information about Afrotherian susceptibility to prion infection *in vivo*. As for our *in vitro* experiments, most Afrotherian species included in this study were unable to misfold in our system, with the exceptions of one sequence belonging to the order Sirenia, the West Indian manatee, as well as some Macroscelidea and Afrosoricida (order within Afrotheria, including small insectivorous mammals like tenrecs from Madagascar and golden moles from Africa) species, which were able to misfold albeit with very low misfolding efficiencies (S3 Fig). In opposition, and as has been noted in nature, most branches belonging to the order Primates included *PRNP* sequences which were successfully misfolded *in vitro*. Therefore, this higher similarity of the *PRNP* of Primates and Afrotheria does not seem to correlate with a certain misfolding propensity. It is worth noting that this similarity between the Afrotherian clade and Euarchontoglires in terms of *PRNP* sequence was also observed in a previous study, where Afrotheria also branched next to the Euarchontoglires clade [55].

The node Boroeutheria (including the remaining placental orders) appeared to be conserved, except for the inclusion of the aforementioned Afrotheria species in the clade, and the exception of the Lagomorpha (order comprising rabbits, hares, and pikas), Hystricomorpha (rodent clade that includes Hystricognathi and other related lineages, such as porcupines and caviomorphs), Erinaceomorpha (formerly used order now often merged into Eulipotyphla; includes hedgehogs and gymnures) and Soricomorpha (outdated grouping formerly including shrews and moles) representative species. The Laurasiatheria node (major placental mammal clade that includes bats, carnivores, ungulates, and insectivores), the next branch in the classical phylogeny, maintained its grand topology at the order level, containing only groups that belong to this taxon according to classical mammalian phylogeny. However, not all the orders were correctly separated. The Eulypotyphla superorder (order of insectivorous mammals including shrews, moles, hedgehogs, and solenodons) presents deviations from classical phylogeny, with one branch representing the Solenodontidae family (primitive family of venomous insectivores native to the Caribbean islands, including the endangered solenodons) of Soricomorpha grouped closer to artiodactyls while representatives from the Soricomorpha Talpidae family (moles and shrews) and two species from the order Erinaceomorpha (hedgehogs) were grouped closer to some representatives of the order Chiroptera (bats). As mentioned, representatives from the family Soricidae (family of shrews, small insectivorous mammals) of Soricomorpha and one species from the Erinaceomorpha (*H. suillus,* the Short-tailed gymnure) are in a cluster that encompasses the most diverse species in terms of *PRNP*. This distribution of the orders does not seem to correlate with the tendency to misfold of the species encompassing these clusters, as they include both sequences that can and cannot misfold, showcasing a misfolding tendency as diverse as the sequences that compose these clusters.

It is worth noting that the Eulypotyphla clade has undergone significant taxonomic revisions over time. Initially part of the now-obsolete Insectivora order, these species were reclassified when Insectivora was found to be paraphyletic [56]. The subsequent orders Erinaceomorpha and Soricomorpha were established, but Soricomorpha was later shown to also be paraphyletic, with Soricidae more closely related to Erinaceidae (family of hedgehogs and gymnures) than to other families in the order [57].

The order Chiroptera (bats) was separated in two groups in the *PRNP* gene tree, representing both existing suborders (Yinpterochiroptera, that includes both large fruit bats and some microbats, and Yangochiroptera, that includes the remaining microbats). Of these, the former was clustered with most Laurasiatheria (major placental mammal clade that includes bats, carnivores, ungulates, and insectivores) and the latter is clustered with some Soricomorpha species as a sister clade of the remaining Laurasiatheria species save the order Carnivora (order of carnivorous mammals, including cats, dogs, bears, seals, and weasels). Notably, the inclusion of the suborder Yinpterochiroptera with most Laurasiatheria, showed a very low bootstrap support (17.6%), indicating uncertainty in this particular grouping. Remarkably, most Yinpterochiroptera are able to misfold *in vitro*, whereas the Yangochiroptera present lower susceptibility to misfolding (S2 Fig), with more proteins being unable to misfold, and those that have generally low misfolding propensities.

Current phylogenies place Eulypotyphla as the first branch of Laurasiatheria, followed by Chiroptera [40], this is not reflected by the *PRNP* gene tree. In the *PRNP* tree, most Laurasiatheria orders were more interwoven than expected by classical phylogeny, Chiroptera was split across two branches and one included Soricomorpha, while other orders maintained, more or less, unique branches. Perissodactyla (odd-toed ungulates, including horses, rhinos, and tapirs) appeared closer to Pholidota (order of pangolins, scale-covered insectivores native to Africa and Asia) than classical phylogeny suggests, and Artiodactyla and Cetacea were grouped together.

The phylogenetic relationship between Artiodactyla and Cetacea remains controversial. Debate continues over whether cetaceans should be considered part of the artiodactyl cluster (rendering the order paraphyletic and leading to the term Cetartiodactyla) or as a closely related monophyletic group. Recent evidence favors the former view [40,58], with cetaceans clustered with hippopotamuses in the Whippopomorpha clade. This arrangement, initially proposed based on genetic evidence [58], is well-supported by our *PRNP* data (Bootstrap 99%). This clustering shows interesting features for misfolding as the Cetacean sequences are unable to spontaneously misfold *in vitro* and the hippopotamuses are among the sequences with the lowest misfolding scores across the artiodactyl species (S3 Fig). The remaining Artiodactyla subgroups (Camelidae, family of camel-like ungulates, including camels, llamas, and alpacas, and Suidae, the pig family, including wild boars and domestic pigs) clustered in intermediate nodes not supported by classic phylogeny (Artiodactyla 3 and 4 subclusters in Fig 2). This divergence from the species tree seems independent from the misfolding proneness of the species grouped together since neither the family Camelidae nor Suidae have characteristic misfolding behaviors that separate them from all other Artiodactyla families.

The order Carnivora was the most different in terms of *PRNP* of all Laurasiatherian orders and the suborders Feliformia (including cats, hyenas, mongooses, and civets) and Caniformia (including dogs, bears, seals, and raccoons) are correctly maintained in terms of *PRNP*.

The node Euarchontoglires was largely conserved, the exclusion of the Lagomorpha order and some representatives of the infraorder Hystricognathi (suborder including porcupines, guinea pigs, capybaras, and other large-bodied South American rodents) of Rodentia as previously mentioned, notwithstanding. However, lower distinctions within this clade showed some divergences from classical phylogeny. The node Glires (a clade including rodents and lagomorphs) was not appreciable, as Lagomorphs were outgrouped from most other Placental mammalian species according to their *PRNP*. This outgrouping does not seem related to differential susceptibility to misfolding according to our previous results [41], although Lagomorphs have traditionally been considered resistant to prion misfolding, in opposition to, the more susceptible, rodents.

As for the remaining orders comprising Euarchontoglires (supraprimate clade including rodents, lagomorphs, primates, tree shrews, and colugos), Primates were separated into three clusters: one including the suborder Strepsirrhini (“wet-nosed” primates, including lemurs, lorises, and galagos), one including the Tarsiiformes (a small group of Southeast Asian primates, the tarsiers) and lastly, one including the suborder Haplorrhini (“dry-nosed” primates, including monkeys, apes, and humans) with the order Dermoptera (colugos or “flying lemurs,” gliding mammals from Southeast Asia). These clusters do not seem to be generated by an evolutionary pressure of prion diseases, as branches that can and cannot misfold are distributed equally throughout the Primates subtree. The separation between these clusters presents varying levels of support, with a very low bootstrap level (38.2%) for the cluster separating Dermoptera from the Haplorrhini. This grouping of Dermoptera with Primates is in agreement with previous studies, which clustered the Dermoptera as a sister clade to Primates [59,60], despite the higher similarity of the *PRNP* of these clades, illustrated by the nesting of Dermoptera within the Primates species. Additional insights on the hominid *PRNP* will be discussed further.

The subsequent cluster grouped Scandentia (order of tree shrews, small arboreal mammals native to Southeast Asia) with the remaining Rodentia cluster. This does not seem to be due to their similar misfolding tendencies, as some rodent species that are correctly clustered within their order show more diverging misfolding results than the order Scandentia compared to the rest of Rodentia. Previous studies have debated whether Scandentia should be grouped with Primates [61], with the Primates+Dermoptera cluster [62], as a sister clade to Glires (Rodentia and Lagomorpha) [63], or as an outgroup to Glires+Primates [64]. In our *PRNP*-based tree, Scandentia appears as a sister clade to most Rodentia species.

The Afrotherians appeared clustered, with Afrosoricida (tenrecs from Madagascar and golden moles from Africa) being the furthest from the others. This group exhibits significant divergence in the *PRNP* phylogeny, deviating from its usual position within Atlantogenata. Notably, Afrotheria is divided into two distinct clusters. One aligns with the group’s traditional topology, encompassing the Macroscelidea order (order of elephant shrews or sengis), supporting the notion that Afrotheria includes some of the most divergent placental mammals according to *PRNP*. The other cluster includes all remaining Afrotheria orders clustered with the Pilosa order (sloths and anteaters) as a sister clade to Euarchontoglires. These deviations from the traditional topology do not seem to be related to misfolding as most Afrotherian species seem to be resistant to misfolding, and those that do misfold are scattered across all three clusters.

The Marsupialia (infraclass of pouched mammals, such as kangaroos, opossums, and koalas) are correctly grouped together by *PRNP*. It is important to note that for some marsupial orders, our sample size is very limited or even non-existent. This paucity of data complicates discrimination, as there are fewer sequences for comparison, increasing the likelihood of erroneous nesting within other orders.

The first to branch off were the Didelphimorphia (order of New World marsupials, including opossums), consistent with the classical phylogeny. The next cluster comprises all other remaining orders, with Dasyuromorphia (order of carnivorous marsupials, such as quolls and the Tasmanian devil) branching away first, in contrast with classical phylogeny where they are the last to branch. The next group to branch off is the Microbiotheria order (a rare South American marsupial order represented by the monito del monte), reminiscent of the position they have in classical phylogeny. Lastly, there is a cluster in which the Diprotodontia branch off a cluster composed of Peramelemorphia (order of bandicoots and bilbies, omnivorous marsupials of Australia) and Notoryctemorphia (the marsupial mole order) reminiscent to classical phylogeny except for the more different in terms of *PRNP* Dasyuromorphia. This grouping of marsupial mammals, while broadly correct, shows some deviations that may be due to limited sampling in some orders or the specific characteristics of the *PRNP* gene in these species.

A peculiar finding among the Marsupialia results is the apparent presence of a nonsense mutation in the coding region of one of the Peramelemorpha sequences (*P. nasuta,* Long-nosed bandicoot) included in this study, potentially resulting in a truncated protein. Since another representative of this order (*M. lagotis,* Greater bilby) was reported with a normal length, it is possible that this is only due to an unsuccessful identification of the whole sequence across contigs in the former sequence. Without access to a sample for independent sequencing, we cannot conclude whether this is a general characteristic of some Peramelemorpha species with significant implications for prion disease research or a limitation of the methodology employed.

In summary, the general topology of the *PRNP* tree closely resembles that of the species tree [40,46] (Fig 2), suggesting that prion diseases have not exerted sufficient evolutionary pressure to significantly alter the evolutionary landscape of the prion protein gene. Moreover, many of the discrepancies we observe occur in branches that have historically been controversial in classical phylogenetic analyses, and do not seem to be related to misfolding proneness as previously discussed. To further investigate the correlation between the *PRNP* gene and propensity to spontaneously form prions, we calculated Fritz & Purvis’ D statistic [65], which assesses the degree of phylogenetic signal in a binary trait. This measure compares the observed trait distribution across phylogeny to two benchmarks: a Brownian motion model of trait evolution, which presents a D value of 0, and a completely random trait distribution, which presents a D value of 1. The Brownian model assumes that traits evolve gradually over time and small changes accumulate from ancestors to descendants, whereas the random model assumes that traits appear in a complete random manner across the phylogenetic tree, independently of the relationship between species. Our analysis, shown in S4 Fig, yielded an estimated D value of 0.304, suggesting an intermediate level of phylogenetic signal. Permutation tests allowed the assessment of the significance of this value, being the probability of observing such D value under a random trait distribution of 0, while under a Brownian model the probability was of 0.007. This supports the interpretation that while there is meaningful phylogenetic structure in the presence of prion misfolding in the tree, it is not fully explained by shared ancestry alone. This agrees with the fact that despite the importance of sequence similarity in prion diseases, distinct residues in a given context can have a greater effect than the overall sequence similarity in misfolding. This result also aligns with the rarity of prion diseases, which have an estimated incidence in humans of only 1–5 individuals per million people per year, predominantly affecting individuals past reproductive age [3]. However, we cannot exclude the possibility that in populations with endemic prion diseases, selective pressure may have acted on the prion protein gene, potentially leading to the emergence of variants that confer resistance to prion diseases, within these species. For instance, a protective variant was described in areas of Papua New Guinea affected by kuru, a human prion disease related to endocannibalistic rituals. In the areas in which the incidence of kuru was very high, a non-synonymous genetic variant was described, rs267606980 (380G > T, Gly127Val), which encodes a valine in codon 127 instead of the more common glycine. This variant has only been described in this region and was present in a high percentage of the susceptible population. Furthermore, it has not been found in kuru patients, and families in which the rs267606980-T allele was present had a significantly lower incidence of kuru [66]. Further studies on the *PRNP* gene have identified that it does not seem to be a rapidly evolving gene, since it shows very few differences among related species such as chimpanzee and human. However, comparing the diversity across human sequences and more distantly related apes and monkeys there seems to be a significant effect of evolution on the human *PRNP* gene, with a greater number of coding variations than would be expected if the gene had not been subjected to selective pressure [48]. For this reason, and despite not having found any specific motifs associated with increased resistance to misfolding, further enquiry of all genetic variants included in the study may yield motifs of interest that have arisen due to selective pressure on the *PRNP* gene of a given species.

### At the core of the branches: From the general topology to differences at nucleotide level in the splits

Following the examination of the tree’s general topology, we analyzed other aspects, particularly the most relevant or interesting nodes. These were selected based on their significance to the tree structure or because they represented sites where the *PRNP* tree topology diverged from classical phylogeny. We then investigated the types of nucleotide variations causing these splits. Nucleotide changes are classified as transversions (purine to pyrimidine or vice versa) or transitions (purine to purine or pyrimidine to pyrimidine). Transitions occur more frequently than expected by chance [67,68], a phenomenon explained by two competing hypotheses: the mutational hypothesis and the selection hypothesis. [69]. To date, neither hypothesis has been definitively proven.

**Fig 3:**
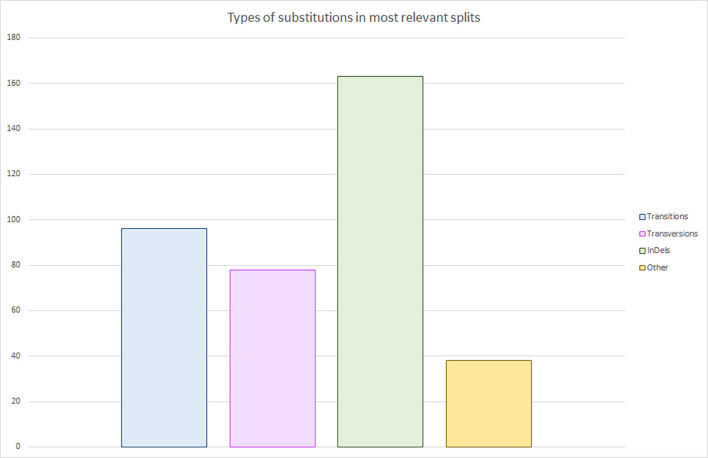
Evaluation of the genetic variations identified in the branch separations of the *PRNP* tree according to its characteristics. The 4 types of DNA variants found in the most relevant splits of the *PRNP* gene tree were evaluated. The most often occurring changes among *PRNP* sequences are insertions and deletions of trinucleotide microsatellites, mostly around the OR region. Next are transitions which are more common than transversions, as described in the literature. Others refers to changes where both transitions and transversions have occurred in one position.

In our analysis of mutations across the *PRNP* tree, we observed a similar trend (Fig 3), with transitions outnumbering transversions across all analyzed splits. However, the most prevalent type of nucleotide variation we encountered were insertions and deletions of trinucleotide microsatellites, primarily in the octapeptide repeated region. This finding is consistent with previous observations of species exhibiting variations from two to seven octapeptide repeats [70]. Looking at the molecular phylogeny of PrP across various vertebrates reveals a noticeable decrease in the variability of repeat units within individual PrP molecules [27]. This could be attributed to processes such as gene conversion and homogenization. Interestingly, as the number of repeated units decreased over evolutionary time, the length of these units increased, culminating in the eight-amino-acid repeats found in mammalian species [71]. Regardless of their evolutionary origin, it is well-established that regions with high codon repetition tend to be unstable and prone to variations that alter the number of repeated units [72]. This instability likely underlies the variable number of octapeptide repeats observed across mammalian species.

### Misfolding across the *PRNP* gene tree: further insights

The phylogenetic analysis of *PRNP* has yielded a wealth of data regarding sequences, similarities, and possible evolutionary events shaping the tree. This study extends beyond phylogenetics to explore the misfolding capacity of these sequences, providing unprecedented insights into the relationship between *PRNP* sequences and their propensity for misfolding. Given that prion strains are conformational variants, understanding how different amino acids affect various conformations can help to elucidate prion misfolding mechanisms and potentially guide the design of prion proteins with controlled characteristics. The relationship between misfolding and the sequence of the prion protein has prompted several studies on *PRNP* phylogeny [73,74]. However, although these studies present a variable number of sequences, none shows a correlation between the *PRNP* sequence and the tendency of the protein to misfold. Although a study from the 1990’s already tried to ascertain the relation between *PRNP* sequence and prion disease susceptibility [75], with the few available mammalian sequences at the time and mostly focused on primates, it was in 2021 when the first systematic study of this sort was carried out [55], where researches compared the available data on natural susceptibility to prion diseases and the *PRNP* sequence. However, as they relied on reports of natural infectivity, the list of sequences available for this study was limited to about 100. In contrast, our previous study [41] analyzes data from over 400 different proteins with varying sequences, assessing their spontaneous misfolding tendencies *in vitro*. This comprehensive dataset, combined with the present phylogenetic analysis, enables a deeper exploration of the correlation between evolutionary PrP sequence variation and misfolding propensity, broadening our understanding beyond previous limitations. It is worth noting that our previous study specifically assessed spontaneous misfolding capacity of all the available PrP variants by PMSA, which may recapitulate mainly the sporadic or spontaneous etiology of prion diseases. Thus, our conclusions may reflect mainly the influence of sporadic prion diseases in *PRNP* evolution, being possibly less significant for scenarios in which acquired prion disorders could have played a role in shaping the evolution of mammals.

In any case, as illustrated in S3 Fig and mentioned throughout this work, there is no apparent correlation between the evolution of the *PRNP* gene and spontaneous misfolding propensity, consistent with previous studies [55]. This lack of correlation suggests that prion diseases, and more specifically, sporadic prion disease, have not significantly shaped the evolution of the *PRNP* gene, as sequences are not clustered by mutations that enhance resistance to spontaneous misfolding. Instead, they are primarily grouped according to their phylogenetic relationships. This observation may seem intuitive, given that prion diseases in humans, for example, typically manifest after reproductive age, thus limiting their evolutionary impact. However, examples from human populations demonstrate that prion diseases can influence *PRNP* gene sequences when individuals are exposed at a young age, as observed with kuru [48]. Furthermore, other prion diseases like scrapie have been shown maternal-to-offspring transmission, with lambs becoming infected mainly through placental tissue [76], through milk suckling [77] and in utero [78]. Early exposure to prion diseases could theoretically lead to selective pressure, as seen in the aforementioned human example. However, one study attempting to determine the effect of prion diseases on the evolution of the sheep *PRNP* gene found that it had not been a major driver of this gene’s evolution in this species. Nevertheless, they did describe an abundance of intraspecies variants and excessive replacement changes which could be indicative of a weak purifying selection, which could indeed have been influenced by prion disease in these populations [79].

In our analysis, *PRNP* sequences that were too similar for the algorithm to discriminate effectively were grouped into previously described consensuses. Notably, these consensuses sometimes include sequences capable of spontaneous misfolding grouped with those that are not, potentially including different genetic variants from the same species (S3 Fig).

While the absence of exclusive folding/misfolding clusters grouped by single nucleotide variations (more often referred to as single nucleotide polymorphisms or SNPs) correlating with resistance or susceptibility to prion diseases suggests that these factors have not significantly shaped the gene’s evolution, there is an effect of sequence on spontaneous misfolding propensity. In fact, our gene tree shows some clusters of resistance and susceptibility where species unable to misfold are grouped together, as are those that can misfold. Nevertheless, these are always supported by the classical phylogeny, indicating that they are presumably not the result of an evolutionary pressure towards sporadic prion disease resistance. This observation is consistent with the notion that the nucleotide sequence is what ultimately determines a given PrP protein’s capacity to misfold into a PrP^Sc^ conformation.

This could be controlled by the ability of certain amino acids to adopt distinct PrP^Sc^ conformations, which is ultimately encoded in the protein’s primary structure. However, this may be a complex issue where even distant residues with no apparent influence on one another in the globular conformation may be highly relevant in the misfolded arrangement.

This study did not uncover a general effect of prion diseases on the evolution of the mammalian *PRNP* gene. However, it has generated a range of data on different *PRNP* sequences and their propensity to misfold spontaneously. By examining the phylogenetic tree, one can identify clusters of reduced misfolding and analyze the common feature they share compared to closely related clusters that exhibit a higher misfolding propensity, as observed between the branches of Diprotodontia (largest order of marsupials, including kangaroos, wallabies, koalas, wombats, possums, and gliders; characterized by two large forward-facing lower incisors). Moreover, it is possible to identify representatives of the same genus where some species can misfold while others show no misfolding, as observed for the Myotis genus (a globally widespread genus of small insectivorous bats known as “mouse-eared bats”). In these cases, since the differences are less pronounced, they could highlight amino acid variants that, in their PrP sequence context, are relevant to the capacity to misfold. In S3 Fig, this can be appreciated by different colors in the tree nodes corresponding to different spontaneous misfolding abilities of distinct variants.

### Zooming in on primates: rs1799990 variant across primate species and time

**Fig 4:**
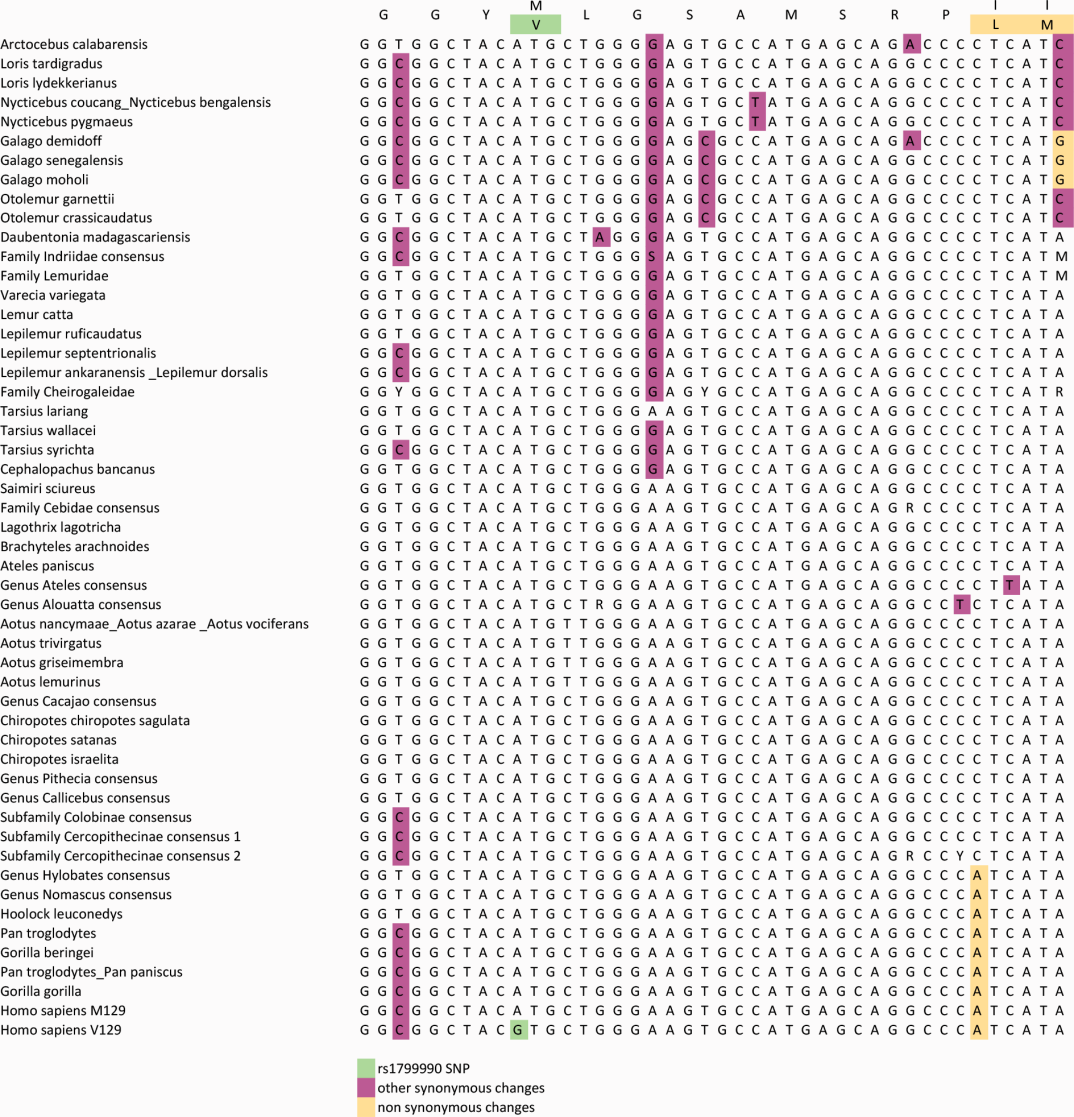
Analysis of the rs1799990 locus in the human PRNP gene compared to other primate’s species included in this study. The variant, highlighted in green, appears only in the human sequence. We have not been able to find it in any other species among all Primates sequenced. The A to G variant causes a non-synonymous change of methionine to valine. Other synonymous changes are highlighted in blue, whereas non-synonymous changes around this region have been highlighted in yellow.

Human *PRNP* variants have been widely studied, and as described previously, some of them can significantly affect prion disease progression. Among these genetic variants, rs1799990 (c.385A > G, p.M129V) stands out, having been extensively studied in relation to several different types of prion diseases [80,81]. Remarkably, the alignment generated in this work did not detect this variant in any other primate sequences analyzed (Fig 4). As shown in Fig 4, this amino acid variant is encoded by an A > G change at the nucleotide level, leading to a methionine-to-valine substitution in the protein. This position is highly conserved in the alignment, and although some sequences exhibit changes, a G at this site has thus far only been detected in *H. sapiens* and marsupials, which have significantly divergent sequences from other mammals in this region. Indeed, when analyzing genomes from ancient human populations, we have found that the G allele was present at a frequency similar to that of modern *H. sapiens* populations since as early as the Chalcolithic (4,500–3,300 BCE), and has been observed, albeit with a slightly lower frequency in samples from the Neolithic (10,000–3,000 BCE). Furthermore, one sample analyzed from the Ust Ishim *H. sapiens,* namely an early modern human living in Siberia approximately 45,000 years ago, was also heterozygous for this genetic variant. This implies that this genetic variant was present in ancient humans. Considering that it has not been detected in other closely related apes, and to further narrow down the dating of the appearance of rs1799990-G allele, we capitalized on the availability of genetic data from Neanderthal and Denisovan individuals generated in the last years to evaluate the presence of this variation in the recent human lineage. Nonetheless, we failed to detect the rs1799990 *locus* in any of the Neanderthal and Denisovan samples analyzed, suggesting it appeared after the divergence of these hominids.

**Fig 5:**
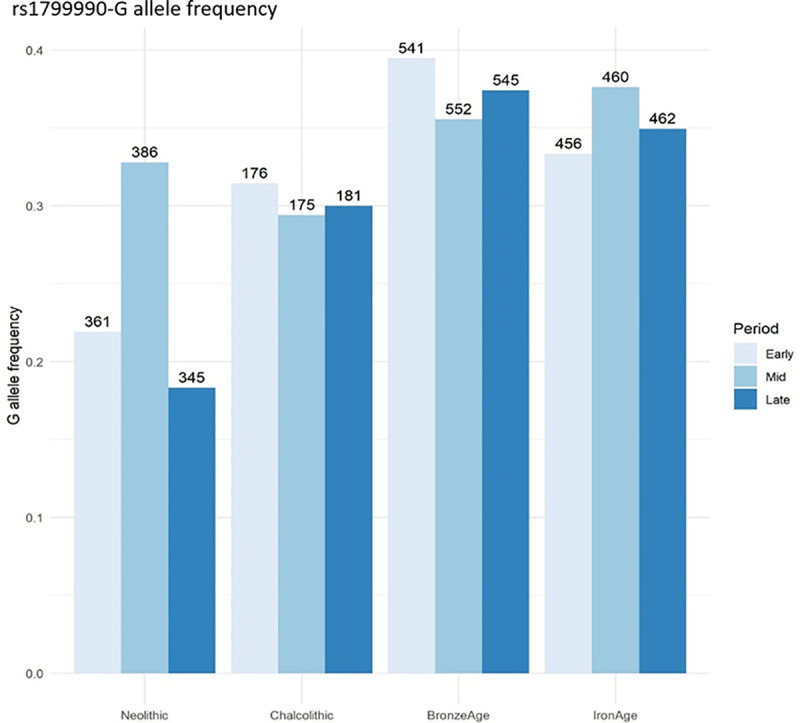
Analysis of rs1799990-G allele frequency in prehistoric human *PRNP.* The rs1799990 variant has been present in human populations as early as the Neolithic with a G-allele frequency of 0.2, which increased to a frequency similar to that of current *H. sapiens* populations (0.3) from the Chalcolithic period and has been maintained around this level throughout the Bronze and Iron Ages until the present.

Therefore, the c.385A > G genetic variant, which has been present in human populations since at least the Upper Paleolithic period (50,000 – 10,000 BCE) (Fig 5), seems unique to *H. sapiens* and marsupials among mammals, the latter of which present markedly differing *PRNP* sequences compared to the rest of mammalian species. Considering the incidence of this variant in modern human populations, its age has been estimated at 1-1.2 million years (Albers and McVean provide an estimate of approximately 39,931 generations in [82], that using standard conversion rates of 25–30 years per generation results in the aforementioned values), preceding the Neanderthal branching. The observed lack of detection could therefore be due to several reasons. It is possible that the variant was present in a common ancestor but lost on the *H. neanderthalensis* branching, while maintained in the *H. sapiens*. Another, perhaps more interesting possibility is that this variant appeared in the latter population due to an evolutionary pressure posed to *H. sapiens* by prion diseases as proposed by Nyström and Hammarström [83] in a similar way to what has been described for the rs267606980 variant and the Fore population, affected by the endemic prion disease known as kuru [66]. Nevertheless, we need to consider that it is possible that we have not had access to enough Neanderthal sequences to detect the less prevalent allele.

Since some variants in the human *PRNP* gene are known to lead to familial forms of prion disease, this gene has been widely sequenced across humans and several sequence variants have been identified. However, few of those have displayed the profound impact of rs1799990 on prion disease [80,81,84]. Some studies indicate that the human *PRNP* gene presents more genetic diversity than expected for a normal evolutionary rate, suggesting evolutionary pressure that forced faster adaptation [48], this would support the theory that it appeared for the first time in early *H. sapiens* rather than having disappeared from the *H. neanderthalensis*. This increased genetic diversity in the human *PRNP* gene has been hypothesized to result from early zoonotic events or, more likely, cannibalism across early *H. sapiens* populations, leading to recycling of prions [48]. In this manner, rs1799990, which confers resistance against some prion disease strains, could have emerged in early *H. sapiens* populations as a protective mechanism against acquired prion disease by ingestion. Evidence of cannibalism across early human populations [85,86] supports a scenario where the selected pressure from cannibalism led to the emergence of prion-resistant variants in the human *PRNP* gene. In fact, some other protective variants have been found in the human PrP that have been associated to prion disease-driven evolutionary selection, such as the variant G127V found among the fore, that suffered the endemic disease of kuru [66] or the variant E219K commonly found in Asian populations and proven to provide protection against vCJD [87]. Similarly, in other mammals suffering from naturally occurring endemic prion diseases such as sheep, several variants with protective effect against scrapie have been described [88].

## Materials and methods

### Extraction and analysis of wild-type and genetic variants of the *PRNP* gene sequences across mammalian species

As TSEs have been documented only in specific mammalian species thus far, our search for *PRNP* genes focused on species from the class Mammalia. We used three different sources: (i) already annotated *PRNP* or prion protein sequences retrieved from GenBank, where we identified 904 sequences corresponding to distinct species. (ii) Sequences extracted from whole genome sequencing projects [Sequence Read Archive (SRA) and DNAZoo], from which we annotated 219 distinct *PRNP* sequences from raw sequencing data. (iii) DNA samples extracted from biological fluid and tissue samples of specimens, namely blood and muscle samples, kindly provided by various zoos and animal shelters. The open reading frame (ORF) of the *PRNP* gene for these species was sequenced in the Molecular (Epi)Genetics Laboratory from Bioaraba Health Research Institute using specifically designed primers based on closely related species with available PrP sequences (S1 Table). This approach allowed us to obtain 23 additional sequences. In total, we compiled 1146 PrP sequences from 901 mammalian species, as the database includes intraspecies variants. As shown in S2 Table, we obtained PrP sequences from representatives of 28 out of 29 orders within the class Mammalia (representing 133 out of 153 families and 457 out of 1231 genera), achieving a robust sample of the variability of PrP sequences across mammals. This collection of sequences is now publicly available in GenBank [National Center for Biotechnology Information (NCBI), accession links for all the sequences are available in S2 Table.

Alignments were carried out using the *PRNP* nucleotide sequences, encompassing the equivalent codons to 23–231 of the bank vole *PRNP* sequence. In these sequences both N-terminal and C-terminal regions corresponding to signal peptides have been removed as they are not part of the mature protein and thus not part of the misfolded PrP. Moreover, as we are expressing the proteins in bacteria that lack the posttranslational modification system of mammalian cells, they would not be removed from the final protein if we included them in the constructs. Consequently, since we aim to study the relationship between the sequence and misfolding, we have decided not to consider the corresponding nucleotides.

### Sequence alignment, curation, and unification of mammalian *PRNP* sequences

The initial dataset consisted of 1146 sequences (see S2 and S3 Tables). These sequences were unified using an *ad hoc* script. This unification process resulted in 1025 unique sequences (S2 Table). In this table, identical sequences are listed under the species names from which they originated but are grouped under a single reference sequence chosen for subsequent misfolding studies. The unique sequences were then aligned using CLUSTALW (https://www.ebi.ac.uk/jdispatcher/msa/clustalo) directly from the MEGAX dashboard [MEGAX: Molecular Evolutionary Genetics Analysis version 11 [89]]. Following this, the alignments were manually curated using this same software. No hypervariable regions were removed; adjustments were primarily made in the octapeptide repeat (OR) region, as different sequences presented a variable number of ORs. The manual curation process mainly involved identifying nucleotides that the alignment software did not align correctly, but which represented the same position. These misalignments were often due to the presence of deletions or insertions in some sequences as well as the variable number of ORs across different species.

### Phylogenetic tree construction and refinement of mammalian *PRNP* sequences: from model selection to consensus building

The best model for the tree was identified using MEGAX as the generalised time reversible (GTR) model [90] with gamma (G) and invariant (I) sites, which estimates a proportion of invariant sites and fits the rest to a gamma distribution [91].

Based on the previously curated alignment of 1025 *PRNP* sequences, a tree was generated using BEAUti and BEAST software [92] under a strict clock using the Yule speciation model, and Monotremanta as root. This tree was obtained using the default parameters, and 10,000,000 iterations were sampled to calculate the bootstrap values, adhering to the developers’ recommended protocol (https://beast.community/first_tutorial). The generated tree was analyzed in Tracer and according to the trace, 10% of the trees generated were discarded as burn-in [92]. The resulting tree presented branches with low bootstrap values (S1 Fig). These branches were identified manually, and where possible, consensus sequences were generated using the BioEdit sequence alignment editor [93]. Consequently, we examined these low bootstrap (less than 50%) nodes and identified biologically relevant consensus sequences, e.g., if they appeared in clusters of sequences from the same genus, or polymorphic variants within one species. The species considered and included in each consensus are illustrated in S4 Table.

The resulting alignment was similarly analysed in MEGAX to identify the best model for the tree which was again GTR + G + I, and a new tree was generated as previously described. This tree, in which most low bootstrap values (less than 50%) were eliminated through the building of consensus sequences, was considered for all further analyses.

### Phylogenetic comparison: tanglegram and Robinson–Foulds distance

To assess the topological congruence between the *PRNP* gene tree and the established species phylogeny, obtained from VertLife [49], we constructed a tanglegram and calculated the Robinson–Foulds (RF) distance [50]. Both trees were rooted and plotted using the R packages *ape* (v5.7) and *phytools* (v1.7). The tanglegram was generated with the cophylo() function from phytools, allowing visual comparison of tip correspondence. The RF distance, a quantitative metric of tree dissimilarity, was calculated using the RF.dist() function from the phangorn package (v2.11.1), which returns the number of differing bipartitions between two unrooted trees. All analyses were conducted in R version 4.4.3.

### Phylogenetic signal: D statistic

To evaluate the phylogenetic signal of prion-related binary traits (e.g., presence/absence of susceptibility), we used Fritz and Purvis’s D statistic [65]. Trait data were assigned to the tips of the species tree, and the statistic was calculated with the *phylo.d()* function from the *caper* package (v1.0.1). This method quantifies the extent to which a binary trait is phylogenetically clustered by comparing observed trait distributions to both random and Brownian motion expectations. Values of D close to 0 indicate Brownian structure, while values near 1 suggest phylogenetic randomness. Simulated null distributions were used to assess significance. All analyses were conducted in R version 4.4.3.

### Assessment of spontaneous misfolding propensity of recombinant PrP variants by PMSA

The spontaneous misfolding capacity of all *PRNP* variants included in this study, was previously assessed by Protein Misfolding Shaking Amplification (PMSA) method. This analysis was previously published in [41] and the methods are comprehensively described there, including a troubleshooting section to facilitate the implementation of the method in other laboratories. Briefly, all different PMSA substrates containing the distinct recombinant PrP variants produced in *E. coli* and purified through IMAC, were disposed in eight 2 ml tubes with screw cap (Fisherbrand), four of them complemented with 100 mg of 1 mm diameter acid-washed glass beads (BioSpec Products, Inc.), and the other four with 100 mg of 0.1 mm diameter acid-washed glass beads (BioSpec Products, Inc.). All eight tubes for each of the substrates were then submitted to PMSA at 39 °C using either a Thermomixer (Eppendorf) or a Digital shaking Drybath (Thermo Scientific) with internal temperature control and shaking at 700 rpm continuously for 24 h. Four serial PMSA rounds were performed for each substrate at dilutions 1:10 and the products of all 4 rounds were digested with proteinase K (PK) (Roche) and visualized through electrophoresis and total protein staining to assess the presence of PK-resistant misfolded rec-PrP (rec-PrP^res^). Depending on the number of rec-PrP^res^ positive tubes and the round of detection, all rec-PrP variants were ranked according to their spontaneous misfolding proneness.

### Phylogenetic tree analysis: the human rs1799990 variant in *H. sapiens* and *H. neanderthalensis*

To investigate genetic variations across different archaeological periods in the human lineage, we analyzsed a comprehensive database with genotype information from 16,389 ancient DNA *H.sapiens* samples (The Allen Ancient DNA Resource (AADR): A curated compendium of ancient human genomes - David Reich Lab Dataverse (harvard.edu)). For our analysis, we selected individuals with a non-zero mean date before 1950 CE, as determined by radiocarbon dating calibrated with OxCal or averaged from contextual dating ranges. This criterion ensured that we included only genuinely ancient samples. After applying this filter, 9624 samples remained. Subsequently, we limited our analysis to samples that were clearly attributed to a specific archaeological period and where each period contained more than 100 samples. This refinement resulted in a total of 4640 samples assigned to the Neolithic, Chalcolithic, Bronze Age, and Iron Age periods, which were further subdivided into Early, Middle, and Late phases based on radiocarbon dating calibrated with OxCal. The distribution of samples within each period, ordered as Early, Middle, and Late, was as follows: 361, 386, and 345 for the Neolithic; 176, 175, and 181 for the Chalcolithic; 541, 552, and 545 for the Bronze Age; and 456, 460, and 462 for the Iron Age. We then extracted genotypes for the variant of interest, rs1799990, and calculated allele frequencies using PLINK v 1.9 (http://pngu.mgh.harvard.edu/purcell/plink/). This approach allowed us to evaluate allele frequencies within specific timeframes and cultural phases, reflecting the chronological context of each group.

## Supporting information

S1 Table List of the 23 species whose *PRNP* has been sequenced in this study together with the forward and reverse primers used to get specific amplicons and phylogenetic family on which primer design was based. (PDF)

S2 Table List of the 1146 *PRNP* sequences, with species classified by their respective orders and arranged alphabetically, that have been analyzed in this study including accession numbers linked to GenBank database (NCBI). For sequences with a great number of known intraspecific variations, one reference sequence has been selected as basal sequence (identified by *) and all others only have the aminoacidic positions that differ from the basal sequence in their common name. The variable positions present in the basal sequence are disclosed in S3 Table. † To identify sequences that differ in their nucleotide sequence but not in their amino acid sequence, which share a species name in the table. Protein nomenclatures instead of nucleotide have been used in this table as many of these variants are known and relevant to the prion community whereas their nucleotide nomenclature may not be as informative. ‡ Whenever a GenBank number is not available, DOI for the reference publication or sample numbers for the whole genome sequences are included.
(PDF)

S1 Text Alignment of the 357 sequences that have been considered for the generation of the *PRNP* gene tree. The final alignment, including all sequences considered in this study, was exported as a.txt, maintaining the gaps along the alignment.
(TXT)

S3 Table List of species for which intraspecies PrP variants were identified, and the amino acid residues that have been considered as wild type throughout the study for each of these species. Variations in these positions are highlighted in the corresponding species in S2 Table.
(PDF)

S1 Fig Prion protein nucleotide sequences based phylogenetic tree compiled using BEAST software. 1146 different nucleotide sequences from 901 different mammalian species were used to perform the analysis as described. Values shown in each branch represent the bootstrap values obtained after 10,000,000 iterations.
(PDF)

S2 Fig Tanglegram comparing *PRNP* gene tree and species tree. A tanglegram showing the topological differences between the *PRNP* gene-based tree and the species consensus tree (generated *ad hoc* using VertLife [49]. Lines connect the same species across both trees, and crossing lines indicate disagreement in tree structure. The Robinson-Foulds (RF) distance [50] between the two trees is 0.52, indicating that approximately 52% of the splits (bipartitions) differ. This suggests moderate topological incongruence between the gene-specific phylogeny and the overall species phylogeny.
(PDF)

S3 Fig *In vitro* misfolding across the *PRNP* phylogenetic tree. The misfolding results have been mapped onto the *PRNP* phylogenetic tree. Stochastic character mapping was used to reconstruct ancestral states and plot them on the nodes of the tree as pies. Blue represents misfolding positive species whereas yellow represent species that have been unable to misfold. The pies fraction of the pie coloured in one or the other represents the fraction of the terminal nodes in which misfolding has been detected or not by PMSA. There is no grouping by prion disease resistant or susceptible amino acids.
(PDF)

S4 Fig Distribution of D statistic under Brownian motion and random trait evolution models. Density plots showing the distribution of the D statistic [65] under two null models: Brownian motion (blue) and random evolution (red), based on 1,000 permutations each. The observed D value for the PRNP misfolding trait is shown as a vertical dashed black line (D = 0.304). The observed value falls closer to the Brownian distribution, suggesting that the trait shows a phylogenetic signal consistent with gradual, tree-structured evolution rather than random distribution. Permutation tests yield p < 0.01 when compared to both null models.
(PNG)

S4 Table List of consensus sequences used in the phylogenetic study and of the species considered to build each consensus sequence. The sequences, identified by their common species names as listed in S2 Table, that are part of each consensus are presented in this table next to the name assigned to each consensus and their corresponding order.
(PDF)

S1 File Excel file including raw data used to generate plots. (XLSX)
