# Supplementary material for: A comprehensive phylogeny of mammalian PRNP gene reveals no influence of prion misfolding propensity on the evolution of this gene
Source: PLoS Pathog. 2025 Jun 25;21(6):e1013257. doi: 10.1371/journal.ppat.1013257 (PMC12208436; doi:10.1371/journal.ppat.1013257)
Supplement: S1 Table — (PDF) [file ppat.1013257.s001.pdf]

Supplementary Table 1. List of the 23 species whose PRNP has been sequenced in this study together with the forward and reverse primers used to get specific amplicons and phylogenetic family on which primer design was based

Species	Forward Primer*	Reverse Primer	Primer based on the family
Lynx lynx	ATGGTGAAAGGCCAC	TCAACCCCCGATCAGG	Felidae
Hylobates muelleri	ATGGCGAACCTTGG	TCATCCCACTATCAGG	Hylobatidae
Mustela lutreola	ATGRTGAAAAGCCACA	TCATCCCACTATCAGG	Mustelidae
Nanger dama	ATGGTGAAAAGCCAC	CTATCCTACTATGAG	Bovidae
Helarctos malayanus	ATGGTGAAAAGCCAC	CCTGATCGTTGGATGA	Ursidae
Callithrix geoffroyi	ATGGCGAACCTTGG	TCATCCCACTATCAGG	Hylobatidae
Dicotyles tajacu	ATGGTGAAAAGCCATATAGG	TCAGCCCACTATGAGG	Suidae
Connochaetes gnou	ATGGTGAAAAGCCAC	CTATCCTACTATGAG	Bovidae
Gazella dorcas	ATGGTGAAAAGCCAC	CTATCCTACTATGAG	Bovidae
Potamochoerus porcus	ATGGTGAAAAGCCATATAGG	TCAGCCCACTATGAGG	Suidae
Nasua nasua	ATGGTGAAAAGCCAC	TCATCCCACTATCAGG	Bovidae (F)/Hylobatidae (R)
Arctocephalus pusillus	ATGGTGAAAAGCCAC	TGGAGAATGAGCAGC	Bovidae (F)/ Otaridae (R)
Vulpes zerda	ATGGTGAAAAGCCAC	AGAATGAGCAGCGAG	Bovidae (F)/Canidae (R)
Macropus rufogriseus	ATGGCAAAAATCCAG	TAGCTCACGATCAGG	Macropodidae
Dromiciops bozinovici	ATGGGAAAAATTCACTTGG	TTAGCTGACAATCAGG	Microbiotheriidae
Phodopus roborovskii	ATGGCGAACCTCAGC	TCATCCCACTATCAGG	Cricetidae (F)/Hylobatidae (R)
Euphractus sexcinctus	ATGGTGAAAAGCCGC	ACCATGAGGAAGACGAG	Chlamyphoridae
Cynomys ludovicianus	ATGGTGARCCCTGG	AGATGAGGAGGATCAC	Sciuridae (F)/Viverridae
Microtus gerbei	ATGGCGAACCTCAGC	TCATCCCACTATCAGG	Cricetidae (F)/Hylobatidae (R)
Eulemur albifrons	ATGGTGAGCCTTGG	AGATGAGGAGGATCAC	Lemuridae (F)/Viverridae (R)
Rupicapra pyrenaica	ATGGTGAAAAGCCAC	CTATCCTACTATGAG	Bovidae
Callithrix argentata	ATGGCAAACCTTGGC	TCATCCCACTATCAGG	Callitrichidae (F)/Hylobatidae (R)
Equus africanus	ATGGTGAAAAGCCAC	AGATGAGGAGGATCAC	Bovidae (F)/Viverridae (R)

***** Degenerated nucleotides have been marked in red, indicating that when designing the primer, there was more than one nucleotide in that position in the family it was based on and therefore a mix of primers was used which included both options for that position (in this case, R = A and G).
