# Supplementary material for: A comprehensive phylogeny of mammalian PRNP gene reveals no influence of prion misfolding propensity on the evolution of this gene
Source: PLoS Pathog. 2025 Jun 25;21(6):e1013257. doi: 10.1371/journal.ppat.1013257 (PMC12208436; doi:10.1371/journal.ppat.1013257)
Supplement: S2 Table — For sequences with a great number of known intraspecific variations, one reference sequence has been selected as basal sequence (identified by *) and all others only have the aminoacidic positions that differ from the basal sequence in their common name. The variable positions present in the basal sequence are disclosed in S3 Table. † To identify sequences that differ in their nucleotide sequence but not in their amino acid sequence, which share a species name in the table. Protein nomenclatures instead of nucleotide have been used in this table as many of these variants are known and relevant to the prion community whereas their nucleotide nomenclature may not be as informative. ‡ Whenever a GenBank number is not available, DOI for the reference publication or sample numbers for the whole genome sequences are included. (PDF) [file ppat.1013257.s002.pdf]

Supplementary Table 2. List of the 1146 PRNP sequences, with species classified by their respective orders and arranged alphabetically, that have been analysed in this study including accession numbers linked to GenBank database (NCBI).

ORDER	BINOMIAL NAME	COMMON NAME	ACCESSION No. (Link) ‡
Afrosoricida	*Amblysomus hottentotus*	Hottentot golden mole	AY133061
	Chrysochloris asiatica	Cape golden mole	XM_006870413
	Echinops telfairi	Lesser hedgehog tenrec	BK064944
	Microgale talazaci	Talazac's shrew tenrec	BK063959
	Tenrec ecaudatus	Tailless tenrec	AY133060
Artiodactyla	*Addax nasomaculatus*	Addax	BK063914
	Aepyceros melampus	Impala	BK063929
	Alces alces	Moose Q109	JQ290077
	Alces alces	Moose R100 I209	MN970212
	Alces alces	Moose I209	AY225485
	Alces alces	Moose*	MN970212
	Alces alces andersonii	Western moose M209	10.4161/pri.19641
	Alces alces andersonii	Western moose I209	10.4161/pri.19641
	Ammotragus lervia	Barbary sheep	EF165080
	Antidorcas marsupialis	Springbok	BK064068
	Antilocapra americana	Pronghorn	AF156187
	Antilope cervicapra	Blackbuck	AY720706
	Axis axis	Axis deer	MT996365
	Axis porcinus	Indian hog deer	BK063963
	Babyrousa celebensis	North Sulawesi babirusa	BK064782
	Beatragus hunteri	Hirola	BK064028
	Bison bison	American bison	AY720696
	Bison bonasus	European bison	EU032298
	Bos frontalis	Gayal	AB534901
	Bos frontalis gaurus	Gaur 6OR	AY720697
	Bos grunniens	Domestic yak H122 V129 S146	AY367635
	Bos grunniens	**Domestic yak 6OR R122 A129 N146**	KC137646
	Bos grunniens mutus	Wild yak 6OR H122 A129 S146	XM_014481732.1
	Bos javanicus	Banteng	AY720691
	Bos javanicus	**Banteng 6OR N185**	AY720693
	Bos taurus	Cow I120	AY247262
	Bos taurus	Cow K218	NM_181015
	Bos taurus	Cow 7OR	AY720445
	Bos taurus	Cow 5OR	AY720525
	Bos taurus	Cow R115 R190	DQ333350
	Bos taurus	Cow†	AY367639
	Bos taurus	Cow R234	AY367642
	Bos taurus	Cow N154	AY367640
	Bos taurus	**Cow***	NM_001271626
	Bos taurus indicus	Zebu 6OR	EU564528
	Bos taurus primigenius	**Aurochs 6OR T106 G108 S154**	D10613
	Bos taurus primigenius	Aurochs S106 S108 N154	D10614
	Boselaphus tragocamelus	Nilgai	AY720700
	Bubalus bubalis	Water buffalo†	AY768533
	Bubalus bubalis	Water buffalo	MK342630
	Bubalus bubalis	**Water buffalo 6OR G108**	AY768534
	Bubalus depressicornis	Anoa Q204	BK063916
	Bubalus depressicornis	Anoa R204	AAV30495
	Budorcas taxicolor	Takin	AB060290
	Camelus bactrianus ferus	Bactrian camel	HQ204566
	Camelus dromedarius	Camel E134	MK655460
	Camelus dromedarius	Camel G134†	MK655461
	Camelus dromedarius	**Camel G134**	Y09760
	Capra falconeri	Markhor	MG214330
	Capra hircus	Goat R101	FJ151011
	Capra hircus	Goat G102	MH567780
	Capra hircus	Goat P110	AF486138
	Capra hircus	Goat T112	DQ149411

Artiodactyla	*Capra hircus*	Goat S127	NM_001314247
	Capra hircus	Goat Q133	DQ345066
	Capra hircus	Goat I137	DQ345067
	Capra hircus	Goat S139	10.1111/j.1365-2052.2009.01873.x
	Capra hircus	Goat F141	10.1371/journal.pone.0061118
	Capra hircus	Goat M142	X91999
	Capra hircus	Goat T142	EF192309
	Capra hircus	Goat R143	AF486135
	Capra hircus	Goat D145	10.1111/j.1365-2052.2009.01873.x
	Capra hircus	Goat D146	10.1016/j.tvjl.2005.09.013
	Capra hircus	Goat S146	10.1111/j.1365-2052.2004.01204.x
	Capra hircus	Goat H151	10.1016/j.tvjl.2005.09.013
	Capra hircus	Goat H154	MH567936
	Capra hircus	Goat Q168	KX611086
	Capra hircus	Goat R106 R171	10.1099/vir.0.017350-0
	Capra hircus	Goat F185	GQ497225
	Capra hircus	Goat P194	EF192310
	Capra hircus	Goat L201	10.1186/1297-9716-42-110
	Capra hircus	Goat T208	10.1016/j.tvjl.2009.10.015
	Capra hircus	Goat Q211	EU032304
	Capra hircus	Goat G211	MH568552
	Capra hircus	Goat R215	10.1371/journal.pone.0061118
	Capra hircus	Goat L218	10.1111/j.1365-2052.2004.01204.x
	Capra hircus	Goat I219	10.1007/s00705-008-0074-1
	Capra hircus	Goat H220	10.1099/0022-1317-83-3-713
	Capra hircus	Goat K222	DQ013244
	Capra hircus	Goat A127 S146	10.1038/s41598-020-63874-z
	Capra hircus	Goat S146 H159	10.1038/s41598-020-63874-z
	Capra hircus	Goat H159	MN795400
	Capra hircus	Goat S146 I193	MN795416
	Capra hircus	Goat I193	MN795423
	Capra hircus	Goat	10.1099/0022-1317-83-3-713
	Capra hircus	**Goat***	XM_005688157
	Capra ibex	Alpine ibex	EF139174
	Capra pyrenaica	Iberian ibex	KT845865
	Capra sibirica	Siberian ibex	BK063969
	Capreolus capreolus	Roe deer	AY639096
	Capreolus pygargus	Siberian roe deer	BK064073
	Capricornis crispus	Japanese serow	MG214326
	Capricornis sumatraensis	Mainland serow	BK068592
	Catagonus wagneri	Chacoan peccary	BK063918
	Cephalophus natalensis harveyi	Harvey's red duiker	BK064031
	Cervus elaphus	Red deer A98 Q226	AY748456
	Cervus elaphus	Red deer A98 S168 Q226	EU032286
	Cervus elaphus	Red deer I208	UFX77122
	Cervus elaphus	**Red deer***	AF016227
	Cervus elaphus	Iberian red deer Q226	KT845863
	Cervus elaphus canadensis	Elk L132	AF016228
	Cervus elaphus canadensis	Elk A191	EU032291
	Cervus elaphus canadensis	**Elk***	EU032294
	Cervus elaphus hanglu	Yarkand deer E226	BK064174
	Cervus nippon	Sika deer G100	EF057409
	Cervus nippon	Sika deer	10.1111/tbed.14543
	Cervus nippon	Sika deer	AY655756
	Connochaetes gnou	Black wildebeest	OR472463
	Connochaetes taurinus	Blue wildebeest	EF165086
	Dama dama	Fallow deer	EF139175
	Damaliscus lunatus	Common tsessebe	BK064037
	Dicotyles tajacu	Collared peccary	OR472462
	Elaphurus davidianus	**Père David's deer S138 I208 E226**	MW804583
	Elaphurus davidianus	Père David's deer N138 M208 Q226	MW804582
	Eudorcas thomsonii	Thomson's gazelle	EU032301
	Gazella dorcas	Dorcas gazelle	OR472464
	Gazella subgutturosa	Goitered gazelle	AF117313
	Giraffa camelopardalis antiquorum	Giraffe 6OR K175	BK064151
	Giraffa camelopardalis reticulata	Reticulata giraffe	AF113942
	Giraffa camelopardalis tippelskirchi	**Masai giraffe 6OR R175**	BK064017

Artiodactyla	*Giraffa camelopardalis tippelskirchi*	Masai giraffe K175	BK068573
	Hemitragus hylocrius	Nilgiri tahr	BK064052
	Hemitragus jayakari	Arabian tahr	MG214331
	Hexaprotodon liberiensis	Pygmy hippopotamus	AB919084
	Hippopotamus amphibius	Hippopotamus	AB919083
	Hippotragus equinus	Roan antelope	BK064055
	Hippotragus niger	Sable antelope L211	EF165085
	Hippotragus niger niger	Sable antelope	BK064056
	Hydropotes inermis	Water deer 5OR G170	10.4172/2157-7579.1000505
	Hydropotes inermis	Water deer 5OR D96	10.4172/2157-7579.1000505
	Hydropotes inermis	Water deer 5OR N100	MK103025
	Hydropotes inermis	Water deer 4OR Q226	MK103026
	Kobus ellipsiprymnus	Waterbuck	EF165087
	Kobus leche leche	Lechwe	BK064060
	Kobus megaceros	Nile lechwe	EF165088
	Lama glama	Llama 6OR	AF113943
	Lama glama cacsilensis	Guanaco	BK064076
	Lama glama chaku	Llama 6OR	BK064075
	Litocranius walleri	Gerenuk	BK064079
	Madoqua kirkii	Kirk's dik-dik	BK064081
	Moschus berezovskii	Dwarf musk deer	BK064096
	Moschus chrysogaster	Alpine musk deer	AY723286
	Moschus moschiferus	Siberian musk deer	BK063923
	Muntiacus crinifrons	Hairy-fronted muntjac	BK064097
	Muntiacus gongshanensis	Gongshan muntjac	BK064177
	Muntiacus muntjak	Indian muntjac	BK064098
	Muntiacus reevesi	Reeves's muntjac	KC788406
	Naemorhedus griseus	Chinese goral	MG214329
	Nanger dama	Dama gazelle	OR472459
	Nanger dama ruficollis	Dama gazelle	BK064170
	Nanger granti	Grant's gazelle	BK064108
	Neotragus moschatus	Suni	BK064111
	Neotragus pygmaeus	Royal antelope	BK064112
	Odocoileus hemionus	Mule deer F225	10.1016/j.gene.2003.10.022
	Odocoileus hemionus	Mule deer G116	AY275712
	Odocoileus hemionus	Mule deer	MT709729
	Odocoileus virginianus	White-tailed deer H95	AY275711
	Odocoileus virginianus	White-tailed deer S96	AF156184
	Odocoileus virginianus	White-tailed deer I103	MG856925
	Odocoileus virginianus	White-tailed deer T123	MG856916
	Odocoileus virginianus	White-tailed deer S96 T123	MG856921
	Odocoileus virginianus	White-tailed deer S96 N100	MG856923
	Odocoileus virginianus	White-tailed deer N100	MG856917
	Odocoileus virginianus	White-tailed deer K226	MN390181
	Odocoileus virginianus	White-tailed deer R226	10.1080/15287390903084264
	Odocoileus virginianus	White-tailed deer T103	MN390064
	Odocoileus virginianus	White-tailed deer V136 R171 S173 N177 I208- Mule deer	MT709729
	Odocoileus virginianus	White-tailed deer T103 H151	MT710148
	Odocoileus virginianus	White-tailed deer R96	MN577936
	Odocoileus virginianus	White-tailed deer H95 S96	AXH06163
	Odocoileus virginianus	White-tailed deer K215	MZ913401
	Odocoileus virginianus	White-tailed deer	XM_020883647
	Odocoileus virginianus clavium	Florida Key deer	MT944346
	Odocoileus virginianus-hemionus	White-tailed deer- Mule deer L230	10.1080/15287390903084264
	Okapia johnstoni	Okapi	BK064032
	Oreamnos americanus	Mountain goat	EF999825
	Oreotragus oreotragus	Klipspringer	BK064025
	Oryx dammah	Scimitar oryx	BK064024
	Oryx gazella	Gemsbok	BK064117
	Ourebia ourebi	Oribi	BK064118
	Ovibos moschatus	Muskox	AF117320
	Ovis ammon	Argali	BK064119
	Ovis aries	Sheep R171	M31313
	Ovis aries	Sheep V136	10.1099/0022-1317-72-10-2411
	Ovis aries	Sheep K176	AF195247
	Ovis aries	Sheep T137	CAA04234
	Ovis aries	Sheep K171	10.1177/0300985818817066

Artiodactyla	*Ovis aries*	Sheep I112	AY304002
	Ovis aries	Sheep 6OR	KF830262
	Ovis aries	Sheep V127	DQ149427
	Ovis aries	Sheep R101	DQ149334
	Ovis aries	Sheep A127 H154	10.1007/s00335-003-2283-y
	Ovis aries	Sheep T136	AJ567987
	Ovis aries	Sheep N138 L189	10.1099/0022-1317-80-9-2527
	Ovis aries	Sheep R138	10.1159/000075731
	Ovis aries	Sheep K142	EF153678
	Ovis aries	Sheep S146 L189	10.1111/j.1365-2052.2004.01204.x
	Ovis aries	Sheep C151	10.1099/0022-1317-80-9-2527
	Ovis aries	Sheep F152	10.1111/j.1365-2052.2004.01204.x
	Ovis aries	Sheep S167	10.1159/000075731
	Ovis aries	Sheep L168	10.1099/vir.0.82083-0
	Ovis aries	Sheep D172	10.1099/vir.0.80047-0
	Ovis aries	Sheep E175	10.1099/vir.0.80047-0
	Ovis aries	Sheep Y180	10.1007/s00335-003-2283-y
	Ovis aries	Sheep H154 L189	DQ677221
	Ovis aries	Sheep H154 R171 R189	EF165079
	Ovis aries	Sheep S195	10.1159/000075731
	Ovis aries	Sheep S196	10.1159/000075731
	Ovis aries	Sheep G151	10.1016/j.livsci.2014.05.005
	Ovis aries	Sheep L189	DQ149335
	Ovis aries	Sheep Q114	10.3390/vetsci10100597
	Ovis aries	Sheep E116	10.3390/vetsci10100597
	Ovis aries	Sheep P118	10.3390/vetsci10100597
	Ovis aries	Sheep V137	10.3390/vetsci10100597
	Ovis aries	Sheep T138	10.3390/vetsci10100597
	Ovis aries	Sheep S145	10.3390/vetsci10100597
	Ovis aries	Sheep K149	10.3390/vetsci10100597
	Ovis aries	Sheep K167	10.3390/vetsci10100597
	Ovis aries	Sheep G169	10.3390/vetsci10100597
	Ovis aries	Sheep H171	10.3390/vetsci10100597
	Ovis aries	Sheep N171	10.3390/vetsci10100597
	Ovis aries	Sheep F192	10.3390/vetsci10100597
	Ovis aries	Sheep M142	HQ197670
	Ovis aries	Sheep H154	AJ000737
	Ovis aries	Sheep T112	AJ000735
	Ovis aries	Sheep S127	AY723289
	Ovis aries	Sheep F141	DQ149343
	Ovis aries	Sheep R143	AF180389
	Ovis aries	**Sheep***	NM_001009481
	Ovis aries orientalis	Moufloun	FJ792606
	Ovis aries vignei	Urial	MG214328
	Ovis canadensis	Bighorn sheep G138 R154	DQ648472
	Ovis canadensis	Bighorn sheep	DQ648473
	Ovis canadensis	Bighorn sheep R154 G210	DQ648476
	Ovis dalli	Dall sheep	DQ648474
	Ovis nivicola lydekkeri	Snow sheep	BK064120
	Pantholops hodgsonii	Tibetan antelope	BK064020
	Phacochoerus africanus	Common warthog	BK063968
	Philantomba maxwellii	Maxwell's duiker	BK064126
	Potamochoerus porcus	Red river hog	OR472465
	Procapra gutturosa	**Mongolian gazelle***	AB473609
	Procapra gutturosa	Mongolian gazelle 6OR S119 G143 H160	AB473614
	Procapra gutturosa	Mongolian gazelle 6OR S119 G143 H160 A172	AB473613
	Procapra gutturosa	Mongolian gazelle 6OR S182 A221	AB473612
	Procapra gutturosa	Mongolian gazelle 6OR	AB473611
	Procapra gutturosa	Mongolian gazelle 6OR S119 I140 G143 H160 A172	AB473615
	Procapra przewalskii	Przewalski's gazelle	BK064131
	Przewalskium albirostris	Thorold's deer	BK064057
	Pseudois nayaur	Bharal	BK064132
	Rangifer tarandus	Reindeer C96 T123 D176	EU032303
	Rangifer tarandus	Reindeer Y225	JQ290076
	Rangifer tarandus	Reindeer S129	DQ154294
	Rangifer tarandus	Reindeer S129 M169	DQ154295
	Rangifer tarandus	Reindeer N138	DQ154292

Artiodactyla	*Rangifer tarandus*	Reindeer D176	JQ290075
	Rangifer tarandus	Reindeer F153 Q226	MT361766
	Rangifer tarandus	Reindeer M207	MW557844
	Rangifer tarandus	Reindeer Q211	MW557845
	Rangifer tarandus	**Reindeer***	AY639093
	Rangifer tarandus caribou	Boreal woodland caribou Q226	SAMN19775459
	Raphicerus campestris	Steenbok	BK064134
	Redunca redunca	Bohor reedbuck	BK064135
	Rucervus eldii thamin	Eld's deer	OL961483
	Rupicapra pyrenaica	Pyrenean chamois 5OR	PQ327921
	Rupicapra pyrenaica	Pyrenean chamois 4OR	KT845868
	Rusa alfredi	Visayan spotted deer	BK068595
	Saiga tatarica	Saiga antelope	BK064016
	Sus scrofa	Pig	L07623
	Sylvicapra grimmia	Common duiker	BK064038
	Syncerus caffer	African buffalo	AY720686
	Taurotragus oryx	Common eland	EF165082
	Tragelaphus angasii	Lowland nyala	EU032296
	Tragelaphus buxtoni	Mountain nyala	BK064065
	Tragelaphus eurycerus	Bongo	BK064137
	Tragelaphus imberbis	Lesser kudu	AY720704
	Tragelaphus scriptus	Harnessed bushbuck	BK063937
	Tragelaphus spekii	Sitatunga	EF165083
	Tragelaphus strepsiceros	Greater kudu A227	X74759
	Tragelaphus strepsiceros	Greater kudu	EF165081
	Tragulus javanicus	Java mouse-deer	BK064004
	Tragulus kanchil	Lesser mouse-deer	BK064005
	Vicugna pacos	Alpaca 5OR	XM_006207354
	Vicugna pacos huacaya	Alpaca	BK064175
	Vicugna vicugna	Vicuna 5OR	BK063925
	Vicugna vicugna mensalis	Vicuña	BK068580

Carnivora	*Acinonyx jubatus*	Cheetah	XM_027069624
	Ailuropoda melanoleuca	Giant panda	XM_034640276
	Ailurus fulgens	Red panda	EU341495
	Aonyx cinerea	Asian small-clawed otter	BK064764
	Arctictis binturong	Binturong	JX218945
	Arctocephalus forsteri	Australasian fur seal	BK064830
	Arctocephalus gazella	Antarctic fur seal	BK064071
	Arctocephalus pusillus	Brown fur seal	OR472468
	Arctocephalus townsendi	Guadalupe fur seal	BK064775
	Bassariscus astutus	Ring-tailed cat	BK064783
	Bassariscus sumichrasti	Cacomistle	BK064784
	Callorhinus ursinus	**Northern fur seal S107**	XM_025876094
	Callorhinus ursinus	Northern fur seal N107	XM_025876616
	Canis adustus	Side-striped jackal	BK064957
	Canis latrans	Coyote	JX218953
	Canis lupus dingo	Dingo	XM_025469395
	Canis lupus familiaris	Dog D163	FJ870767
	Canis lupus familiaris	Dog G101 D163	KY649554
	Canis lupus familiaris	Dog G101	KY649558
	Canis lupus familiaris	**Dog***	KY649563
	Canis mesomelas	Black-backed jackal	BK064772
	Caracal caracal	Caracal	BK063932
	Catopuma temminckii	Asian golden cat	JX218947
	Chrysocyon brachyurus	Maned wolf	JX218978
	Crocuta crocuta	Spotted hyena	JX218985
	Cryptoprocta ferox	Fossa	BK063948
	Cystophora cristata	Hooded seal	JX218976
	Eira barbara	Tayra	BK064178
	Enhydra lutris	Sea otter	XM_022494297
	Enhydra lutris kenyoni	Sea otter	BK063955
	Enhydra lutris nereis	Sea otter	BK063956
	Erignathus barbatus	Bearded seal	BK064769
	Eumetopias jubatus	Steller sea lion	EU341498
	Felis chaus	Jungle cat	BK064169

Carnivora	*Felis nigripes*	Black-footed cat	BK064047
	Felis silvestris catus	Cat	EU588730
	Genetta genetta	Common genet	OR963560
	Gulo gulo	Wolverine	EU341500
	Gulo gulo luscus	Wolverine	BK068578
	Halichoerus grypus	Grey seal	BK064050
	Helarctos malayanus	Sun bear	OR472460
	Helogale parvula	Common dwarf mongoose	BK064051
	Herpestes javanicus	Javan mongoose	EU341501
	Herpestes javanicus auropunctatus	Small Indian mongoose	BK068565
	Hyaena hyaena	Striped hyena	BK064018
	Leopardus geoffroyi	Geoffroy's cat	BK064182
	Leopardus tigrinus	Oncilla	BK064829
	Leopardus wiedii	Margay	JX218979
	Leptonychotes weddellii	Weddell seal	XM_006744312
	Lontra canadensis	North American river otter	XM_032859119
	Lutra lutra	European otter	JX218963
	Lycaon pictus	African wild dog	BK064019
	Lynx canadensis	Canada lynx	XM_030310019
	Lynx lynx	Eurasian lynx	OR472456
	Lynx pardinus	Iberian lynx	BK064941
	Lynx rufus	Bobcat	EU341503
	Martes flavigula	Yellow-throated marten	BK064787
	Martes foina	Beech marten	BK064788
	Martes martes	European pine marten	JX218964
	Martes pennanti	Fisher	BK068567
	Martes zibellina	Sable	BK063939
	Meles meles	European badger	JX218960
	Mellivora capensis	Honey badger	BK064009
	Mephitis mephitis	Skunk	EU341504
	Mirounga angustirostris	Northern elephant seal	BK064093
	Mirounga leonina	Southern elephant seal	XM_035005902
	Monachus schauinslandi	Hawaiian monk seal	XM_021689157
	Mungos mungo	Banded mongoose	BK064099
	Mustela erminea	Stoat	EU341505
	Mustela lutreola	European mink	OR472458
	Mustela nigripes	Black-footed ferret	BK064188
	Mustela nivalis	Least weasel	BK064157
	Mustela putorius	Ferret M113 L179	10.1099/0022-1317-75-11-2947
	Mustela putorius	**Ferret I113 F179**	BK064943
	Nasua narica	White-nosed coati	BK064789
	Nasua nasua	South American coati	OR472467
	Neofelis diardi	Sunda clouded leopard	BK064215
	Neofelis nebulosa	Clouded leopard	EU341506
	Neovison vison	American mink	EF508270
	Nyctereutes procyonoides	Raccoon dog	EU341507
	Odobenus rosmarus	**Walrus**	EU341508
	Odobenus rosmarus divergens	Walrus	BK064116
	Otaria flavescens	Sea lion	JX218981
	Otocolobus manul	Pallas's cat	BK064223
	Otocyon megalotis	Bat-eared fox	BK064006
	Pagophilus groenlandicus	Harp seal	JX218971
	Paguma larvata	Masked palm civet	BK064230
	Panthera leo	Lion	EU236260
	Panthera onca	Jaguar	JX218977
	Panthera pardus	Leopard	XM_019462612
	Panthera tigris	**Siberian tiger**	OR472455
	Panthera tigris jacksoni	Malayan tiger	BK064160
	Panthera tigris sumatrae	Sumatran tiger	JX218987
	Panthera uncia	Snow leopard	BK064798
	Paradoxurus hermaphroditus	Asian palm civet	BK063947
	Phoca largha	Spotted seal	BK064792
	Phoca vitulina	Common seal	EU341509
	Potos flavus	Kinkajou	BK063964
	Prionailurus bengalensis euptilurus	Leopard cat	BK064130
	Prionailurus iriomotensis	Iriomote cat	BK064141
	Prionailurus viverrinus	Fishing cat	BK064149

Carnivora	*Procyon lotor*	Raccoon	EU341510
	Proteles cristata	Aardwolf	BK064008
	Pteronura brasiliensis	Giant otter	BK064061
	Puma concolor	Cougar	XM_025932838
	Puma yagouaroundi	Jaguarundi	BK064172
	Pusa hispida saimensis	Ringed seal	BK064218
	Pusa sibirica	Baikal seal	BK064225
	Speothos venaticus	Bush dog	JX218950
	Spilogale gracilis	Western spotted skunk	BK064000
	Spilogale putorius interrupta	Eastern spotted skunk	BK064191
	Suricata suricatta	Meerkat	XP_029772432
	Taxidea taxus jeffersonii	American badger	BK064062
	Tremarctos ornatus	Spectacled bear	BK064152
	Urocyon cinereoargenteus	Gray fox	BK064774
	Urocyon littoralis	Island fox	BK064773
	Ursus americanus	American black bear	EU341512
	Ursus arctos	Brown bear	JX218961
	Ursus maritimus	Polar bear	BK063942
	Ursus thibetanus japonicus	Asian black bear	BK063943
	Ursus thibetanus thibetanus	Asian black bear	BK063926
	Vulpes corsac	Corsac fox	MN381732
	Vulpes ferrilata	Tibetan fox	BK068579
	Vulpes lagopus	Arctic fox	EU365392
	Vulpes velox	Swift fox	EU341513
	Vulpes vulpes	Red fox	XM_026012328
	Vulpes zerda	Fennec fox	OR472469
	Zalophus californianus	California sea lion	XM_027623145

Cetacea	*Balaenoptera acutorostrata*	Common minke whale	XM_007191701
	Balaenoptera bonaerensis	Antarctic minke whale	BK064027
	Balaenoptera borealis	Sei whale	BK064828
	Balaenoptera edeni	Bryde's whale	BK064766
	Balaenoptera musculus	Blue whale	BK064013
	Balaenoptera physalus	Whale	DQ884475
	Balaenoptera ricei	Rice's whale	BK064217
	Caperea marginata	Pygmy right whale	BK064226
	Cephalorhynchus commersonii	Commerson's dolphin	BK064767
	Delphinapterus leucas	Beluga whale	XM_022556485
	Delphinus delphis	Common dolphin	DQ884473
	Eschrichtius robustus	Gray whale	BK064015
	Eubalaena australis	Southern right whale	BK064770
	Eubalaena glacialis	North Atlantic right whale	BK064771
	Eubalaena japonica	North Pacific right whale	BK064042
	Feresa attenuata	Pygmy killer whale	AB919061
	Globicephala melas	Long-finned pilot whale	XM_030872630
	Grampus griseus	Risso's dolphin	DQ884471
	Hyperoodon ampullatus	Northern bottlenose whale	BK064204
	Inia geoffrensis	Amazon river dolphin	BK064059
	Kogia breviceps	Pygmy sperm whale	AB919063
	Kogia sima	Dwarf sperm whale	AB919064
	Lagenorhynchus acutus	Atlantic white-sided dolphin	BK064776
	Lagenorhynchus albirostris	White-beaked dolphin	BK064229
	Lagenorhynchus obliquidens	Pacific white-sided dolphin	XM_027118243
	Lipotes vexillifer	Baiji	XM_007463977
	Megaptera novaeangliae	Humpback whale	BK064082
	Mesoplodon bidens	Sowerby's beaked whale	BK064083
	Mesoplodon densirostris	Blainville's beaked whale	BK064778
	Mesoplodon europaeus	Gervais' beaked whale	BK064779
	Mesoplodon stejnegeri	Stejneger's beaked whale	BK064780
	Monodon monoceros	Narwhal	XM_029221065
	Neophocaena phocaenoides	Indo-Pacific finless porpoise A234 S235	AB919066
	Neophocaena phocaenoides asiaeorientalis	**Narrow-ridged finless porpoise A234 S235**	BK068574
	Neophocaena phocaenoides asiaeorientalis	Narrow-ridged finless porpoise Y234 G235	XM_024764249
	Orcinus orca	Orca Y234 G235	XM_004276438
	Orcinus orca	**Orca A234 S235**	XM_012535450
	Peponocephala electra	Melon-headed whale	AB919067

Cetacea	*Phocoena phocoena*	Harbour porpoise	AB919069
	Phocoena sinus	**Vaquita 3OR A234 S235**	XP_032461337
	Phocoena sinus	Vaquita Y234 G235	XM_032605442
	Phocoenoides dalli	Dall's porpoise	AB919068
	Physeter catodon	Sperm whale	XM_007123285
	Platanista gangetica	South Asian river dolphin	BK064007
	Platanista minor	Indus river dolphin	BK064144
	Pontoporia blainvillei	La Plata dolphin 6OR	BK068576
	Pontoporia blainvillei	La Plata dolphin	BK064129
	Pseudorca crassidens	False killer whale	BK068598
	Sousa chinensis	Indo humpback dolphin	BK064136
	Stenella attenuata	Pantropical spotted dolphin	BK064799
	Stenella clymene	Clymene dolphin	BK064800
	Stenella coeruleoalba	Dolphin	DQ884470
	Stenella frontalis	Atlantic spotted dolphin	BK064801
	Stenella longirostris	Spinner dolphin	AB919073
	Steno bredanensis	Rough-toothed dolphin	DQ884468
	Tursiops aduncus	Indo bottlenose dolphin	AB919075
	Tursiops truncatus	Common bottlenose dolphin K184	DQ130070
	Tursiops truncatus	Common bottlenose dolphin N184 Y234 G235	XM_019941481
	Tursiops truncatus	**Common bottlenose dolphin A234 S235**	XM_004324888
	Ziphius cavirostris	Ziphius	DQ884467

Chiroptera	*Anoura caudifer*	Tailed tailless bat	BK064067
	Antrozous pallidus	Pallid bat	BK064069
	Artibeus jamaicensis	Jamaican fruit bat	BK064072
	Artibeus lituratus	Great fruit-eating bat	BK068594
	Aselliscus stoliczkanus	Stoliczka's trident bat	BK067940
	Carollia perspicillata	Seba's short-tailed bat	BK063935
	Corynorhinus rafinesquii	Rafinesque's big-eared bat	BK064814
	Corynorhinus townsendii	Townsend's big-eared bat	BK064211
	Craseonycteris thonglongyai	Kitti's hog-nosed bat	BK063940
	Cynopterus brachyotis	Lesser short-nosed fruit bat	BK063952
	Cynopterus sphinx	Indian short-nosed fruit bat	AY133046
	Desmodus rotundus	Common vampire bat	BK064947
	Eidolon dupreanum	Madagascan fruit bat	BK064786
	Eidolon helvum	Straw-coloured fruit bat	BK064039
	Eonycteris spelaea	Cave nectar bat	BK064041
	Eptesicus fuscus	Big brown bat	BK064945
	Eptesicus nilssonii	Northern bat	BK064950
	Glossophaga soricina mutica	Pallas's long-tongued bat	BK068597
	Hipposideros armiger	Great roundleaf bat	XM_019644637
	Hipposideros galeritus	Cantor's roundleaf bat	BK064054
	Hipposideros larvatus	Intermediate roundleaf bat	BK067864
	Hipposideros turpis pendleburyi	Pendlebury's roundleaf bat	BK064185
	Ia io	Great evening bat	BK064208
	Lasiurus borealis	Eastern red bat	BK064077
	Lasiurus cinereus	Hoary bat	BK063928
	Macroglossus sobrinus	Long-tongued fruit bat	BK063991
	Macrotus californicus	California leaf-nosed bat	AY133049
	Megaderma lyra	Greater false vampire bat	BK064080
	Micronycteris hirsuta	Hairy big-eared bat	BK064088
	Miniopterus natalensis	Natal long-fingered bat	XM_016212061
	Miniopterus schreibersii	Common bent-wing bat	BK064092
	Molossus alvarezi	Alvarez's mastiff bat	BK064952
	Molossus molossus	Velvety free-tailed bat	KAF6472180
	Molossus rufus	Northern black mastiff bat	BK064213
	Mormoops blainvillei	Antillean ghost-faced bat	BK063931
	Murina aurata feae	Little tube-nosed bat	BK064100
	Myotis brandtii	Brandt's bat	XM_005874678
	Myotis daubentonii	Daubenton's bat	AY133048
	Myotis davidii	David's myotis	XM_006758058
	Myotis findleyi	Findley's bat	BK068596
	Myotis lucifugus	Little brown bat	XM_006096427
	Myotis myotis	Greater mouse-eared bat	BK064107
	Myotis nattereri	Natterer's bat	BK068607

Chiroptera	*Myotis planiceps*	Flat-headed myotis	BK068610
	Myotis ricketti	Rickett's big-footed bat	BK064146
	Myotis septentrionalis	Northern long-eared bat	BK064803
	Myotis vivesi	Fish-eating bat	BK068582
	Myotis yumanensis	Yuma myotis	BK064222
	Noctilio leporinus	Greater bulldog bat	BK064113
	Nyctalus aviator	Birdlike noctule	BK068590
	Nycticeius humeralis	Evening bat	BK064115
	Phyllostomus discolor	Pale spear-nosed bat Δ38-47 N160	XM_036009593
	Phyllostomus discolor	Pale spear-nosed bat 6OR N160	XM_028524368
	Phyllostomus discolor	**Pale spear-nosed bat***	RXPB02009858
	Phyllostomus hastatus	Greater spear-nosed bat	BK064158
	Pipistrellus kuhlii	Kuhl's pipistrelle 4OR	XM_036417932
	Pipistrellus kuhlii	**Kuhl's pipistrelle 5OR**	XM_036417931
	Pipistrellus pipistrellus	Common pipistrelle 5OR	BK068575
	Pipistrellus pipistrellus	**Common pipistrelle 4OR**	BK064127
	Plecotus auritus	Lump-nosed bat	OR472453
	Pteronotus parnellii	Parnell's mustached bat	BK063967
	Pteropus alecto	Black flying fox	XM_015597302
	Pteropus medius	Indian flying fox	XM_039863787
	Pteropus pselaphon	Bonin flying fox	BK063965
	Pteropus rodricensis	Rodrigues flying fox	BK064817
	Pteropus rufus	Madagascan flying fox	BK064809
	Pteropus vampyrus	Large flying fox	XM_011369934
	Rhinolophus affinis	Intermediate horseshoe bat	BK068599
	Rhinolophus ferrumequinum	Greater horseshoe bat	BK063922
	Rhinolophus hipposideros	Lesser horseshoe bat	BK068604
	Rhinolophus sinicus	Chinese rufous horseshoe bat	BK063992
	Rhynchonycteris naso	Greater horseshoe bat	BK064953
	Rousettus aegyptiacus	Egyptian fruit bat S102 V178	JACASE010000012.1
	Rousettus aegyptiacus	**Egyptian fruit bat N102 I178**	XM_016135122
	Rousettus leschenaultii	Leschenault's rousette	BK063997
	Rousettus madagascariensis	Madagascan rousette	BK064805
	Saccopteryx bilineata	Greater sac-winged bat	BK068589
	Saccopteryx leptura	Lesser sac-winged bat	BK068591
	Sturnira ludovici hondurensis	Honduran yellow-shouldered bat	BK063970
	Tadarida brasiliensis	Mexican free-tailed bat	BK064001
	Tonatia saurophila	Stripe-headed round-eared bat	BK064003
	Trachops cirrhosus	Fringe-lipped bat	BK064958
	Vespertilio murinus	Parti-coloured bat	BK068585
Cingulata	*Cabassous unicinctus*	Southern naked-tailed armadillo	BK064228
	Chaetophractus vellerosus	Screaming hairy armadillo	BK064063
	Dasypus novemcinctus	Nine-banded armadillo	EU559337
	Euphractus sexcinctus	Six-banded armadillo	OR472476
	Tolypeutes matacus	Southern three-banded armadillo	BK064002
Dasyuromorphia	*Antechinus flavipes*	Yellow-footed antechinus	BK063971
	Antechinus stuartii	Brown antechinus	BK063930
	Dasyurus hallucatus	Northern quoll	BK064760
	Dasyurus viverrinus	Eastern quoll	BK064810
	Myrmecobius fasciatus	Numbat	BK064811
	Phascogale calura	Red-tailed phascogale	BK064832
	Phascogale tapoatafa	Brush-tailed phascogale	BK064816
	Sarcophilus harrisii	Tasmanian devil	XM_003757949
	Sminthopsis crassicaudata	Fat-tailed dunnart	BK064171
	Thylacinus cynocephalus	Thylacine	BK064189
Dermoptera	*Cynocephalus volans*	Philippine flying lemur	BK064216
	Galeopterus variegatus	Sunda flying lemur	AY133034
Didelphimorphia	*Didelphis virginiana*	Virginia opossum	BK064768
	Gracilinanus agilis	Agile gracile opossum	BK063958
	Monodelphis domestica	Opossum S153	NM_001040028

	Monodelphis domestica	Opossum N153	FR728385
Diprotodontia	*Acrobates pygmaeus*	Feathertail glider	BK068568
	Bettongia penicillata ogilbyi	Woylie	BK064808
	Cercartetus concinnus	Western pygmy possum	BK064813
	Dendrolagus matschiei	Matschie's tree-kangaroo	BK064823
	Distoechurus pennatus	Feather-tailed possum	BK068569
	Gymnobelideus leadbeateri	Leadbeater's possum	BK064074
	Lagorchestes hirsutus	Rufous hare-wallaby	BK064777
	Macropus eugenii	Wallaby	AY659988
	Macropus fuliginosus	Western grey kangaroo	BK064820
	Macropus giganteus	Eastern grey kangaroo	BK064781
	Macropus irma	Western Brush Wallaby	BK064824
	Macropus robustus	Common wallaroo	BK064831
	Macropus rufogriseus	Red-necked wallaby	OR472470
	Macropus rufus	Red kangaroo	OR472471
	Petauroides volans	Southern greater glider	BK068571
	Petaurus breviceps	Sugar glider	BK064219
	Petrogale xanthopus	Yellow-footed rock-wallaby	OR472466
	Phalanger gymnotis	Ground cuscus	BK064791
	Phascolarctos cinereus	Koala	XM_020985342
	Potorous gilbertii	Gilbert's potoroo	BK064793
	Pseudocheirus peregrinus occidentalis	Western ringtail	BK064794
	Pseudochirops archeri	Green ringtail possum	BK068570
	Pseudochirops corinnae	Plush-coated ringtail possum	BK064795
	Pseudochirops cupreus	Coppery ringtail possum	BK064796
	Setonix brachyurus	Quokka	BK064765
	Trichosurus vulpecula	Common brushtail possum	L38993
	Vombatus ursinus	Common wombat	XM_027865324
	Wallabia bicolor	Swamp wallaby	BK064825
Erinaceomorpha	*Atelerix albiventris*	Four-toed hedgehog	OR472473
	Erinaceus europaeus	European hedgehog	EU572708
	Hylomys suillus	Short-tailed gymnure	AY133044
Hyracoidea	*Heterohyrax brucei*	Yellow-spotted rock hyrax	BK064053
	Procavia capensis	Rock hyrax	AY133057
Lagomorpha	*Lepus americanus*	Snowshoe hare	BK063972
	Lepus europaeus	Brown hare	OR472454
	Lepus oiostolus	Woolly hare	BK068583
	Lepus timidus	Mountain hare	BK063973
	Lepus townsendii	White-tailed jackrabbit	BK064227
	Ochotona curzoniae	Plateau pika	MT371369
	Ochotona princeps	American pika	EU555402
	Oryctolagus cuniculus	Rabbit	NM_001082021
	Sylvilagus bachmani	Brush rabbit	BK063974
	Sylvilagus floridanus	Eastern cottontail	BK064951
	Sylvilagus transitionalis	New England cottontail	BK064962
Macroscelidea	*Elephantulus edwardii*	Cape elephant shrew	XM_006894222
	Macroscelides proboscideus	Round-eared elephant shrew	AY133059
Microbiotheria	*Dromiciops bozinovici*	Pancho's colocolo opossum	OR472474
	Dromiciops gliroides	Colocolo opossum	BK064161
Monotremata	*Ornithorhynchus anatinus*	Platypus	XM_029065640
	Tachyglossus aculeatus	Short-beaked echidna	BK063950

Notoryctemorphia	*Notoryctes typhlops*	Southern marsupial mole	BK064812
Peramelemorphia	*Macrotis lagotis*	Greater bilby	BK068593
	Perameles nasuta	Long-nosed bandicoot	BK068609
Perissodactyla	*Ceratotherium simum cottoni*	Northern white rhinoceros	BK063975
	Ceratotherium simum simum	Southern white rhinoceros	XM_014790100
	Dicerorhinus sumatrensis harrissoni	Bornean rhinoceros	BK063994
	Dicerorhinus sumatrensis sumatrensis	Bornean rhinoceros	BK063995
	Diceros bicornis	Black rhinoceros	AY133052
	Diceros bicornis minor	Black rhinoceros	BK063976
	Equus africanus	African wild ass	OR472483
	Equus asinus	Asinus M211	FJ746570
	Equus asinus	**Asinus V211**	BK064942
	Equus burchellii	Burchell's zebra	EF165074
	Equus caballus ferus	Horse K175	10.1080/19336896.2018.1513316
	Equus caballus ferus	Horse G134 C165	EU887254
	Equus caballus ferus	Horse V68 D183	EU887256
	Equus caballus ferus	Horse N233	EU887258
	Equus caballus ferus	Horse R101	10.1038/s41598-020-65731-5
	Equus caballus ferus	**Horse***	EU887260
	Equus caballus przewalskii	**Przewalski's horse***	XM_008519788.1
	Equus caballus przewalskii	Przewalski's horse C130	EU887250
	Equus caballus przewalskii	Przewalski's horse I111	EU887245
	Equus grevyi	Grévy's zebra	BK064961
	Equus kiang	Kiang	EF165076
	Equus quagga boehmi	Grant's zebra	AF117329
	Equus zebra hartmannae	Mountain zebra	EF165073
	Rhinoceros unicornis	White rhinoceros	BK064948
	Tapirus bairdii	Baird's tapir	BK064815
	Tapirus indicus	Malayan tapir	BK063977
	Tapirus terrestris	South American tapir	BK063978
Pholidota	*Manis crassicaudata*	Indian pangolin	BK063961
	Manis culionensis	Philippine pangolin	BK067868
	Manis gigantea	Giant pangolin	BK067865
	Manis javanica	Pangolin	XM_017663893
	Manis pentadactyla	Chinese pangolin	BK063962
	Manis temminckii	Temminck's pangolin	BK067866
	Manis tetradactyla	Long-tailed pangolin	BK067867
	Manis tricuspis	Tree pangolin	BK063960
Pilosa	*Bradypus variegatus*	Brown-throated sloth	BK063934
	Choloepus didactylus	Linnaeus's two-toed sloth	BK068572
	Choloepus didactylus	Linnaeus's two-toed sloth	BK063945
	Choloepus hoffmanni	Hoffmann's two-toed sloth	BK063946
	Cyclopes didactylus 4OR	Silky anteater	AY133063
	Cyclopes didactylus 5OR	Silky anteater	AY133062
	Myrmecophaga tridactyla	Giant anteater	BK063979
	Tamandua tetradactyla	Southern tamandua	BK063980
Primates	*Allenopithecus nigroviridis*	Allen's swamp monkey	BK064763
	Alouatta belzebul	Red-handed howler	AY765382
	Alouatta caraya	Black howler	BK067917
	Alouatta discolor	Spix's red-handed howler	BK067937
	Alouatta juara	Juruá red howler	BK067938
	Alouatta macconnelli	Guyanan red howler	BK067939
	Alouatta palliata	Mantled howler	BK064066
	Alouatta seniculus puruensis	Colombian red howler	BK067929
	Aotus azarae	Azara's night monkey	BK067883
	Aotus griseimembra	Gray-handed night monkey	BK067884
	Aotus lemurinus	Gray-bellied night monkey	AY765387

Primates	*Aotus nancymaae*	Nancy Ma's night monkey	XM_012455393
	Aotus trivirgatus	Three-striped night monkey	U08293
	Aotus vociferans	Spix's night monkey	BK067885
	Arctocebus calabarensis	Calabar angwantibo	BK067992
	Ateles belzebuth	White-bellied spider monkey	BK067886
	Ateles chamek	Peruvian spider monkey	BK067887
	Ateles fusciceps	Black-headed spider monkey	BK068588
	Ateles geoffroyi	Geoffroy's spider monkey	U08309
	Ateles hybridus	Brown spider monkey	BK064173
	Ateles marginatus	White-cheeked spider monkey	BK067941
	Ateles paniscus	Red-faced spider monkey	U15164
	Avahi laniger	Eastern woolly lemur	BK067888
	Avahi peyrierasi	Peyrieras's woolly lemur	BK067869
	Brachyteles arachnoides	Southern muriqui	AY765383
	Cacajao ayresi	Aracá uakari	BK067889
	Cacajao calvus	Bald uakari	AY765381
	Cacajao hosomi	Neblina uakari	BK067890
	Cacajao melanocephalus	Golden-backed uakari	BK067891
	Callicebus bernhardi	Prince Bernhard's titi monkey	BK067942
	Callicebus brunneus	Brown titi monkey	BK067922
	Callicebus caligatus	Chestnut-bellied titi monkey	BK067928
	Callicebus cinerascens	Ashy black titi monkey	BK067915
	Callicebus cupreus	Coppery titi monkey	BK068605
	Callicebus donacophilus	White-eared titi	BK064142
	Callicebus dubius	Hershkovitz's titi monkey	BK067904
	Callicebus grovesi	Alta Floresta titi monkey	BK067914
	Callicebus hoffmannsi	Hoffmanns's titi monkey	BK067943
	Callicebus lucifer	Lucifer titi monkey	BK067894
	Callicebus lugens	Black titi monkey	BK067895
	Callicebus miltoni	Milton's titi monkey	BK067944
	Callicebus moloch	Red-bellied titi	U08312
	Callicebus torquatus	Collared titi monkey	BK067896
	Callimico goeldii	Goeldi's marmoset	AY765390
	Callithrix argentata	Silvery marmoset	OR472482
	Callithrix geoffroyi	White-headed marmoset	OR472461
	Callithrix humeralifer	Santarem marmoset	BK067945
	Callithrix humilis	Roosmalens' dwarf marmoset	BK067946
	Callithrix jacchus	Common marmoset S96 K219	U08304
	Callithrix jacchus	**Common marmoset N96 R219**	XM_008995703
	Callithrix kuhlii	Wied's marmoset	BK067892
	Callithrix pygmaea	Pygmy marmoset	AY765392
	Cebus albifrons	Humboldt's white-fronted capuchin	BK064029
	Cebus apella	Tufted capuchin L48	U08295
	Cebus apella	**Tufted capuchin R48**	XM_032295507
	Cebus capucinus	Panamanian white-faced capuchin	XM_017524036
	Cebus capucinus imitator	Panamanian white-faced capuchin	BK064030
	Cebus olivaceus	Wedge-capped capuchin	BK067947
	Cebus unicolor	Spix's white-fronted capuchin	BK067948
	Cephalopachus bancanus	Horsfield's tarsier	BK064214
	Cercocebus atys	Sooty mangabey D144	XM_012052159
	Cercocebus atys	Sooty mangabey	XM_012052159
	Cercocebus chrysogaster	Golden-bellied mangabey	BK067936
	Cercocebus torquatus	Collared mangabey	U75385
	Cercopithecus albogularis	Sykes' monkey	BK064193
	Cercopithecus ascanius	Red-tailed monkey	BK067949
	Cercopithecus campbelli lowei	Campbell's mona monkey	BK067924
	Cercopithecus cephus	Moustached guenon	BK067950
	Cercopithecus diana	Guenons	U08292
	Cercopithecus hamlyni	Hamlyn's monkey	BK067951
	Cercopithecus lhoesti	L'Hoest's monkey	BK067952
	Cercopithecus mitis	Blue monkey	BK067994
	Cercopithecus mona	Mona monkey	U75386
	Cercopithecus neglectus	DeBrazza's monkey	U75387
	Cercopithecus petaurista	Lesser spot-nosed monkey	BK067953
	Cercopithecus pogonias	Crested mona monkey	BK067930
	Cercopithecus preussi	Preuss's monkey	BK067954
	Cercopithecus roloway	Roloway monkey	BK067955

Primates	*Cercopithecus solatus*	Sun-tailed monkey	BK067956
	Cheirogaleus major	Greater dwarf lemur	BK067893
	Cheirogaleus medius	Fat-tailed dwarf lemur	BK063936
	Chiropotes chiropotes sagulata	Red-backed bearded saki	BK067897
	Chiropotes israelita	Brown-backed bearded saki	BK067898
	Chiropotes satanas	Black bearded saki	AY765380
	Chlorocebus aethiops	Grivet	U08291
	Chlorocebus pygerythrus	Vervet monkey	BK067957
	Chlorocebus sabaeus	Green monkey	XM_008019068
	Colobus angolensis palliatus	Angola colobus 3OR	XM_011941046
	Colobus angolensis palliatus	Angola colobus 2OR	XM_011941047
	Colobus angolensis palliatus	Angola colobus	XM_011941048
	Colobus guereza	Mantled guereza	U75389
	Colobus polykomos	King colobus	BK067958
	Daubentonia madagascariensis	Aye-aye	BK064012
	Erythrocebus patas	Patas monkey	U75388
	Erythrocebus patas	Patas monkey†	BK068563
	Eulemur albifrons	White-headed lemur	OR472479
	Eulemur collaris	Collared brown lemur	BK067993
	Eulemur coronatus	Crowned lemur	BK067870
	Eulemur fulvus	Common brown lemur	BK064044
	Eulemur macaco	Black lemur	BK064045
	Eulemur macaco flavifrons	Blue-eyed black lemur	BK064043
	Eulemur mongoz	Mongoose lemur	BK064802
	Eulemur rubriventer	Red-bellied lemur	BK067899
	Eulemur rufifrons	Red-fronted lemur	BK068608
	Eulemur rufus	Red lemur	BK067871
	Eulemur sanfordi	Sanford's brown lemur	BK067872
	Galago demidoff	Prince Demidoff's bushbaby	BK067901
	Galago moholi	Mohol bushbaby	BK064194
	Galago senegalensis	Senegal bushbaby	BK067900
	Gorilla beringei	Eastern gorilla	BK067933
	Gorilla gorilla	Western gorilla	U08300
	Hapalemur alaotrensis	Lac Alaotra bamboo lemur	BK067874
	Hapalemur griseus	Eastern lesser bamboo lemur	BK067873
	Hapalemur meridionalis	Southern lesser bamboo lemur	BK067875
	Hapalemur occidentalis	Western lesser bamboo lemur	BK067876
	Homo sapiens	Human V129	BC012844
	Homo sapiens	**Human M129**	NM_001080123
	Hoolock leuconedys	Eastern hoolock gibbon	BK064201
	Hylobates agilis	Agile gibbon	BK064785
	Hylobates klossii	Kloss's gibbon	BK067959
	Hylobates lar	Lar gibbon	U08299
	Hylobates moloch	Silvery gibbon	XM_032143110
	Hylobates muelleri	Müller's gibbon	OR472457
	Hylobates pileatus	Pileated gibbon	BK064186
	Indri indri	Indri	BK064058
	Lagothrix lagotricha	Brown woolly monkey	AY765384
	Lemur catta	Ring-tailed lemur	BK064078
	Leontopithecus chrysomelas	Golden-headed lion tamarin	BK064822
	Leontopithecus rosalia	Golden lion tamarin	BK063917
	Lepilemur ankaranensis	Ankarana sportive lemur	BK067877
	Lepilemur dorsalis	Gray-backed sportive lemur	BK067878
	Lepilemur ruficaudatus	Red-tailed sportive lemur	BK067879
	Lepilemur septentrionalis	Northern sportive lemur	BK067880
	Lophocebus aterrimus	Black crested mangabey	U75384
	Loris lydekkerianus	Gray slender loris	BK067902
	Loris tardigradus	Red slender loris	BK064198
	Macaca arctoides	Stump-tailed macaque	U08311
	Macaca assamensis	Assam macaque	EF455529
	Macaca cyclopis	Formosan rock macaque	BK064212
	Macaca fascicularis	Crab-eating macaque C151	EU311599
	Macaca fascicularis	Crab-eating macaque	U08298
	Macaca fuscata	Japanese macaque	U08301
	Macaca leonina	Northern pig-tailed macaque	BK067960
	Macaca maura	Moor macaque	BK067961
	Macaca mulatta	Rhesus macaque E52 S97 N100	AY382293

Primates	*Macaca mulatta*	Rhesus macaque Q52 N97 H100	NM_001047152
	Macaca nemestrina	Pigtail macaque	U08306
	Macaca nemestrina	Pigtail macaque	XM_011741222
	Macaca nigra	Celebes crested macaque	BK067860
	Macaca radiata	Bonnet macaque	BK067921
	Macaca siberu	Siberut macaque	BK067962
	Macaca silenus	Lion-tailed macaque	BK064196
	Macaca sylvanus	Barbary macaque	U75382
	Macaca thibetana thibetana	Tibetan macaque	BK067862
	Macaca tonkeana	Tonkean macaque	BK067963
	Mandrillus leucophaeus	Drill 2OR	XM_011975232
	Mandrillus leucophaeus	Drill 3OR	XM_011975225
	Mandrillus leucophaeus	Drill 4OR	XM_011975206
	Mandrillus leucophaeus	Drill Δ117-141	XM_011975229
	Mandrillus leucophaeus	**Drill 6OR**	XM_011975232
	Mandrillus sphinx	Mandrill	U08303
	Microcebus griseorufus	Reddish-gray mouse lemur	BK064084
	Microcebus mittermeieri	Mittermeier's mouse lemur	BK064085
	Microcebus murinus	Gray mouse lemur	NM_001309920
	Microcebus ravelobensis	Golden-brown mouse lemur	BK064086
	Microcebus tavaratra	Northern rufous mouse lemur	BK064087
	Miopithecus ogouensis	Gabon talapoin	BK067934
	Miopithecus talapoin	Angolan talapoin	BK064818
	Mirza coquereli	Coquerel's giant mouse lemur	BK064094
	Mirza zaza	Northern giant mouse lemur	BK064095
	Nasalis larvatus	Proboscis monkey	BK064109
	Nomascus annamensis	Northern buffed-cheeked gibbon	BK067964
	Nomascus concolor	Black crested gibbon	BK067916
	Nomascus gabriellae	Yellow-cheeked gibbon	BK067965
	Nomascus leucogenys	N white-cheeked gibbon	XM_012511857
	Nomascus siki	S white-cheeked gibbon	BK064199
	Nycticebus bengalensis	Bengal slow loris	BK064202
	Nycticebus coucang	Sunda slow loris	BK064114
	Nycticebus pygmaeus	Pygmy slow loris	OR472481
	Otolemur crassicaudatus	Brown greater galago 6OR	BK068564
	Otolemur garnettii	Northern greater galago	XM_003788036
	Pan paniscus	Bonobo	BK064946
	Pan troglodytes	Chimpanzee 4OR	GABF01007620
	Pan troglodytes	Chimpanzee 5OR	U08296
	Papio anubis	Olive baboon	XM_031656585
	Papio cynocephalus	Yellow baboon	BK067966
	Papio hamadryas	Hamadryas baboon	U08294
	Papio papio	Guinea baboon	BK064790
	Papio ursinus	Chacma baboon	BK067927
	Perodicticus potto	West African potto	BK067903
	Piliocolobus badius	Western red colobus	BK067968
	Piliocolobus gordonorum	Udzungwa red colobus	BK067969
	Piliocolobus kirkii	Zanzibar red colobus	BK067970
	Piliocolobus tephrosceles	Ugandan red colobus	XM_023217934
	Pithecia albicans	White-footed saki	BK067971
	Pithecia chrysocephala	Golden-faced saki	BK067972
	Pithecia hirsuta	Hairy saki	BK067973
	Pithecia irrorata	Rio Tapajós saki	AY765379
	Pithecia mittermeieri	Mittermeier's Tapajós saki	BK067974
	Pithecia pissinatti	Pissinatti's saki	BK067975
	Pithecia pithecia	White-faced saki	BK064128
	Pithecia vanzolinii	Vanzolini's bald-faced saki	BK067976
	Pongo abelii	Sumatran orangutan	XM_009233395
	Pongo pygmaeus	Bornean orangutan	U08305
	Presbytis comata	Javan surili	BK067977
	Presbytis melalophos mitrata	Black-crested Sumatran langur	BK067918
	Prolemur simus	Greater bamboo lemur	BK063921
	Propithecus coquereli	Coquerel's sifaka	XM_012639270
	Propithecus deckenii coronatus	Von der Decken's sifaka	BK067905
	Propithecus diadema	Diademed sifaka	BK067906
	Propithecus edwardsi	Milne-Edwards's sifaka	BK067907
	Propithecus perrieri	Perrier's sifaka	BK067908

Primates	*Propithecus tattersalli*	Golden-crowned sifaka	BK067881
	Propithecus verreauxi	Verreaux's sifaka	BK067909
	Pygathrix cinerea	Gray-shanked douc	BK067978
	Pygathrix nemaeus	Red-shanked douc	BK064133
	Pygathrix nigripes	Black-shanked douc	BK064197
	Rhinopithecus bieti	Black snub-nosed monkey	XM_017878075
	Rhinopithecus roxellana	Golden snub-nosed monkey	BK063920
	Rhinopithecus strykeri	Myanmar snub-nosed monkey	BK064200
	Saguinus bicolor	Pied tamarin	AY765388
	Saguinus fuscicollis	Brown-mantled tamarin	BK067923
	Saguinus imperator	Emperor tamarin	BK064034
	Saguinus inustus	Mottle-faced tamarin	BK067979
	Saguinus labiatus	White-lipped tamarin	BK067980
	Saguinus midas	Golden-handed tamarin	BK064187
	Saguinus mystax	Moustached tamarin	BK067981
	Saguinus nigricollis	Black-mantled tamarin	BK067920
	Saguinus oedipus	Cotton-top tamarin	BK067863
	Saimiri boliviensis	Black-capped squirrel monkey	BK064035
	Saimiri cassiquiarensis	Humboldt's squirrel monkey	BK067910
	Saimiri macrodon	Ecuadorian squirrel monkey	BK067911
	Saimiri oerstedii	Central American squirrel monkey	BK067912
	Saimiri sciureus	Squirrel monkey K164	JN811553
	Saimiri sciureus	Squirrel monkey 5OR K164	U15165
	Saimiri sciureus	Squirrel monkey R164	U08310
	Saimiri ustus	Bare-eared squirrel monkey	BK067913
	Semnopithecus entellus	Northern plains gray langur	BK064033
	Semnopithecus hypoleucos	Black-footed gray langur	BK067919
	Semnopithecus priam	Tufted gray langur	BK067982
	Semnopithecus schistaceus	Nepal gray langur	BK067983
	Symphalangus syndactylus	Siamang	U08308
	Tarsius lariang	Lariang tarsier	BK067984
	Tarsius syrichta	Philippine tarsier	XM_021710036
	Tarsius wallacei	Wallace's tarsier	BK067985
	Theropithecus gelada	Gelada 4OR	U75383
	Theropithecus gelada	Gelada	XM_025399776
	Trachypithecus auratus	East Javan langur	BK067932
	Trachypithecus cristatus	Silvery lutung	BK067986
	Trachypithecus francoisi	François' langur 5OR	U08302
	Trachypithecus francoisi	François' langur 4OR	XM_033185731
	Trachypithecus germaini	Germain's langur	BK067935
	Trachypithecus hatinhensis	Hatinh langur	BK067987
	Trachypithecus johnii	Nilgiri langur	BK067988
	Trachypithecus laotum	Laotian langur	BK067989
	Trachypithecus obscurus	Dusky leaf monkey	BK067931
	Trachypithecus phayrei crepuscula	Phayre's leaf monkey	BK064195
	Trachypithecus pileatus	Capped langur	BK067925
	Trachypithecus poliocephalus	Cat Ba langur	BK067926
	Trachypithecus vetulus	Purple-faced langur	BK067990
	Varecia rubra	Red ruffed lemur	BK067882
	Varecia variegata	Black-and-white ruffed lemur	BK067861
Proboscidea	*Elephas maximus*	Asian elephant	AY133055
	Loxodonta africana	Elephant	EU588731
Rodentia	*Abrothrix hirta*	Long-haired grass mouse	BK064176
	Abrothrix longipilis hirtus	Long-haired akodont	BK064147
	Acomys cahirinus	Cairo spiny mouse	EF467171
	Acomys kempi	Kemp's spiny mouse	BK068600
	Acomys percivali	Percival's spiny mouse	BK068601
	Allactaga bullata	Gobi jerboa	BK064143
	Allactaga sibirica	Mongolian five-toed jerboa	BK068587
	Aplodontia rufa	Mountain beaver	BK064070
	Apodemus fulvipectus	Steppe field mouse	KF466953
	Apodemus mystacinus	E broad-toothed field mouse	KF466942
	Apodemus speciosus	Large Japanese field mouse	BK064145

Rodentia	*Apodemus sylvaticus*	Wood mouse	AF367623
	Arvicanthis niloticus	African grass rat	XM_034493892
	Arvicola amphibius	European water vole	BK063953
	Bandicota indica	Greater bandicoot rat	KF466950
	Bandicota savilei	Savile's bandicoot rat	KF466951
	Bathyergus suillus	Cape dune mole-rat	BK068566
	Berylmys berdmorei	Small white-toothed rat	KF466946
	Capromys pilorides	Desmarest's hutia	BK064046
	Castor canadensis	North American beaver	XM_020184027
	Cavia aperea	Brazilian guinea pig	KM357834
	Cavia porcellus	Guinea pig	XM_003476602
	Cavia tschudii	Montane guinea pig	BK063938
	Chinchilla lanigera	Long-tailed chinchilla	XM_005380818
	Chiropodomys gliroides	Indo pencil-tailed tree mouse	KF466945
	Coendou prehensilis	Brazilian porcupine	OR472472
	Cricetomys ansorgei	Southern giant pouched rat	BK064819
	Cricetomys gambianus	Gambian pouched rat	BK063941
	Cricetulus barabensis	Chinese hamster	M33958
	Cricetulus migratorius	Armenian hamster	M33959
	Cryptomys damarensis	Damaraland mole-rat	XM_010607544
	Cryptomys darlingi	Mashona mole-rat	BK064181
	Ctenodactylus gundi	Common gundi	BK063949
	Ctenomys sociabilis	Social tuco-tuco	BK063951
	Cynomys gunnisoni	Gunnison's prairie dog	BK064140
	Cynomys ludovicianus	Black-tailed prairie dog	OR472477
	Dasyprocta punctata	Central American agouti	BK063998
	Dicrostonyx torquatus	Arctic lemming	BK064224
	Dinomys branickii	Pacarana	BK063954
	Dipodomys merriami	Merriam's kangaroo rat 6OR	BK068577
	Dipodomys merriami	Merriam's kangaroo rat 5OR	BK064205
	Dipodomys ordii	Ord's kangaroo rat	XM_013010955
	Dipodomys spectabilis	Banner-tailed kangaroo rat	BK064155
	Dipodomys stephensi	Stephens' kangaroo rat	BK064064
	Dipus sagitta	Northern three-toed jerboa	BK068584
	Dolichotis patagonum	Patagonian mara	KM357833
	Ellobius lutescens	Transcaucasian mole vole	BK064014
	Ellobius talpinus	Northern mole vole	BK064040
	Eospalax fontanierii	Chinese zokor	BK064949
	Erethizon dorsata	North American porcupine	BK063957
	Geomys bursarius	Plains pocket gopher	BK068603
	Glaucomys volans	Southern flying squirrel	BK064183
	Glis glis	Edible dormouse	BK064048
	Grammomys surdaster	African woodland thicket rat	XM_028757107
	Graphiurus murinus	Woodland dormouse	BK064049
	Heterocephalus glaber	Naked mole-rat	XM_004840771
	Hydrochoerus hydrochaeris	Capybara	KM357831
	Hydromys chrysogaster	Rakali	BK064827
	Hylomyscus alleni	Allen's wood mouse	BK064167
	Hystrix brachyura	Malayan porcupine	BK063933
	Hystrix cristata	Crested porcupine	BK063944
	Jaculus jaculus	Lesser Egyptian jerboa	BK064939
	Kerodon rupestris	Rock cavy	KM357832
	Lagostomus maximus	Plains viscacha	BK064960
	Leopoldamys edwardsi	Edwards's long-tailed giant rat	KF466944
	Leopoldamys sabanus	Long-tailed giant rat	KF466943
	Lophiomys imhausi	Maned rat	BK064153
	Marmota flaviventris	Yellow-bellied marmot	XM_027955665
	Marmota himalayana	Himalayan marmot	BK063993
	Marmota marmota	**Alpine marmot 2OR**	XM_015482632
	Marmota marmota marmota	Alpine marmot 2OR†	BK063988
	Marmota monax	Groundhog	XM_046433852
	Marmota vancouverensis	Vancouver Island marmot	BK063989
	Mastacomys fuscus	Broad-toothed mouse	BK064762
	Mastomys coucha	Southern multimammate mouse	XM_031371966
	Mastomys natalensis	Natal multimammate mouse	BK064165
	Maxomys surifer	Red spiny rat	KF466947
	Meriones libycus	Libyan jird	BK064826

Rodentia	*Meriones meridianus*	Midday jird	BK068586
	Meriones unguiculatus	Mongolian gerbil	XM_021651076
	Mesocricetus auratus	Syrian hamster	AH001830
	Mesocricetus brandti	Turkish hamster	EU886369
	Microtus agrestis	Field vole	AF367625
	Microtus arvalis	Common vole	BK064089
	Microtus californicus	California vole	BK064221
	Microtus fortis	Reed vole	BK064090
	Microtus gerbei	Gerbe's vole	OR472478
	Microtus montanus	Montane vole	BK064179
	Microtus ochrogaster	Prairie vole	XM_005365568
	Microtus oeconomus	Tundra vole	BK064091
	Microtus oregoni	Creeping vole	BK064150
	Microtus pennsylvanicus	Meadow vole	GQ850541
	Microtus richardsoni	Water vole	BK064156
	Microtus richardsoni arvicoloides	Water vole†	BK064180
	Mus caroli	Ryukyu mouse	XM_021185802
	Mus cervicolor	Fawn-colored mouse	KF466939
	Mus cookii	Cook's mouse	KF466940
	Mus fragilicauda	Sheath-tailed mouse	KF466956
	Mus minutoides	African pygmy mouse	BK064101
	Mus musculus	Mouse F108 V189	M18071
	Mus musculus	**Mouse L108 T189**	NM_011170
	Mus musculus castaneus	House mouse	BK064102
	Mus musculus domesticus	House mouse	BK064104
	Mus musculus musculus	House mouse	BK064103
	Mus pahari	Gairdner's shrewmouse	XM_021192940
	Mus spicilegus	Steppe mouse	BK063924
	Mus spretus	Algerian mouse	BK064105
	Muscardinus avellanarius	Hazel dormouse	BK064106
	Myocastor coypus	Coypu	BK064010
	Myodes gapperi	Southern red-backed vole	GQ850538
	Myodes glareolus	Bank vole M109	AF367624
	Myodes glareolus	Bank vole I109	PQ327920
	Myodes rutilus	Northern red-backed vole	BK068602
	Neodon shergylaensis	Shergylaensis vole	BK064207
	Neotoma lepida	Desert woodrat	BK064110
	Octodon degus	Common degu	XM_023718589
	Octomys mimax	Mountain viscacha rat	BK064011
	Ondatra zibethicus	Muskrat	BK064026
	Onychomys torridus	Southern grasshopper mouse	XM_036185260
	Pachyuromys duprasi	Fat-tailed gerbil	BK067991
	Pedetes capensis	South African springhare	BK064021
	Perognathus longimembris pacificus	Little pocket mouse	BK064022
	Peromyscus attwateri	Texas mouse	BK064023
	Peromyscus aztecus	Aztec mouse	BK064121
	Peromyscus californicus insignis	California mouse	BK064122
	Peromyscus crinitus	Canyon mouse	BK064807
	Peromyscus eremicus	Cactus mouse	BK064123
	Peromyscus leucopus	White-footed mouse	XM_028878611
	Peromyscus maniculatus bairdii	Deer mouse	XM_006983992
	Peromyscus maniculatus sonoriensis	Deer mouse	BK068581
	Peromyscus melanophrys	Plateau mouse	BK064124
	Peromyscus mexicanus	Mexican deer mouse	BK064125
	Peromyscus nasutus	Northern rock mouse	BK064804
	Peromyscus polionotus subgriseus	Oldfield mouse	EF467170
	Petaurista alborufus	Red and white giant flying squirrel	KM357835
	Petromus typicus	Dassie rat	BK063990
	Phodopus campbelli	Campbell's dwarf hamster	EU886367
	Phodopus roborovskii	Roborovski hamster	OR472475
	Phodopus sungorus	Russian hamster	EU886368
	Phyllotis vaccarum	Punta de Vacas leaf-eared mouse	BK067967
	Praomys delectorum	Delectable soft-furred mouse	BK064166
	Psammomys obesus	Fat sand rat	BK063966
	Pseudomys desertor	Desert mouse	BK064761
	Pseudomys fumeus	Smoky mouse	BK064821
	Rattus argentiventer	Ricefield rat	KF466955

Rodentia	*Rattus exulans*	Polynesian rat	KF466949
	Rattus losea	Lesser ricefield rat	KF466954
	Rattus nitidus	Himalayan field rat	KF466952
	Rattus norvegicus	Brown rat	BK063913
	Rattus rattus	Rat	XM_032904254
	Rattus tanezumi	Tanezumi rat	KF466948
	Rhabdomys dilectus	Mesic four-striped grass rat	BK064164
	Rhabdomys pumilio	Four-striped grass mouse	BK064232
	Rhizomys pruinosus	Hoary bamboo rat	BK063999
	Rhombomys opimus	Great gerbil	BK063996
	Rhynchomys soricoides	Mount Data shrew-rat	BK064163
	Salpingotus crassicauda	Thick-tailed pygmy jerboa	BK068606
	Saxatilomys paulinae	Paulina's limestone rat	KF466958
	Sciurus carolinensis	Eastern gray squirrel	BK064159
	Sciurus lis	Japanese squirrel	FN678794
	Sciurus niger	Fox squirrel	BK064184
	Sciurus stramineus	Guayaquil squirrel	KM357836
	Sciurus vulgaris	Red squirrel	AY133037
	Sigmodon fulviventer	Tawny-bellied cotton rat	AF117324
	Sigmodon hispidus	Hispid cotton rat	BK064036
	Spalax ehrenbergi	Middle East blind mole-rat	AY133041
	Spalax galili	Upper Galilee Mountains blind mole-rat	XM_008835893
	Spermophilus beecheyi	California ground squirrel	BK064192
	Spermophilus dauricus	Daurian ground squirrel	BK063919
	Spermophilus parryii	Arctic ground squirrel	XM_026385776
	Spermophilus tridecemlineatus	Thirteen-lined ground squirrel	XM_005320503
	Tamias sibiricus	Siberian chipmunk	BK064209
	Thomomys bottae	Botta's pocket gopher	BK064206
	Thryonomys swinderianus	Greater cane rat	BK064162
	Tokudaia muenninki	Muennink's spiny rat	BK064955
	Tokudaia osimensis	Ryukyu spiny rat	BK064210
	Tokudaia tokunoshimensis	Tokunoshima spiny rat	BK064956
	Tympanoctomys barrerae	Plains viscacha rat	BK064138
	Typhlomys cinereus	Chinese pygmy dormouse	BK064190
	Uromys caudimaculatus	Giant white-tailed rat	BK064806
	Xerus inauris	Cape ground squirrel	BK064148
	Xerus rutilus	Unstriped ground squirrel	BK064797
	Zapus hudsonius	Meadow jumping mouse	BK064139
Scandentia	*Tupaia belangeri chinensis*	Chinese tree shrew	XM_006163978
	Tupaia tana	Large treeshrew	AY133035
Sirenia	*Dugong dugon*	Dugong	BK063981
	Hydrodamalis gigas	Steller's sea cow	BK063982
	Trichechus manatus	West Indian manatee 6OR	AY133056
	Trichechus manatus latirostris	West Indian manatee 6OR	BK063983
Soricomorpha	*Condylura cristata*	Star-nosed mole	XM_012728373
	Crocidura russula	Greater white-toothed shrew	OR472480
	Galemys pyrenaicus	Pyrenean desman	BK063927
	Scalopus aquaticus	Eastern mole	BK063985
	Solenodon paradoxus woodi	Hispaniolan solenodon	BK063984
	Sorex araneus	Common shrew	BK064940
	Sorex cinereus	Cinereous shrew	BK063915
	Sorex fumeus	Smoky shrew	BK064954
	Sorex maritimensis	Maritime shrew	BK064231
	Sorex palustris	American water shrew	BK064220
	Suncus etruscus	Etruscan shrew	BK064203
	Talpa europaea	European mole	AY133042
	Talpa occidentalis	Spanish mole	BK063986
Soricomorpha	*Uropsilus gracilis*	Gracile shrew mole	BK063987
Tubulidentata	*Orycteropus afer*	Aardvark	AY133058

* For sequences with a great number of known polymorphic variations, one reference sequence has been selected as basal sequence (identified by *) and all others only have the aminoacidic positions that differ from the basal sequence in their common name. The polymorphic positions present in the basal sequence are disclosed in Supplementary table 3.

† To identify sequences that differ in their nucleotide sequence but not in their amino acid sequence, which share a species name in the table. Protein nomenclature instead of nucleotide has been used in this table as many of these polymorphisms are known and relevant to the prion community whereas their nucleotide nomenclature may not be as informative.

‡ Whenever a GenBank number is not available, DOI or sample numbers for the whole genome sequences are included.
