# Supplementary material for: A comprehensive phylogeny of mammalian PRNP gene reveals no influence of prion misfolding propensity on the evolution of this gene
Source: PLoS Pathog. 2025 Jun 25;21(6):e1013257. doi: 10.1371/journal.ppat.1013257 (PMC12208436; doi:10.1371/journal.ppat.1013257)
Supplement: S3 Table — Variations in these positions are highlighted in the corresponding species in S2 Table. (PDF) [file ppat.1013257.s004.pdf]

Supplementary Table 3. List of species in which polymorphic PrP variants were identified, and the amino acid residues that have been considered as wild type throughout the study for each of these species Variations in these polymorphic positions are highlighted in species in Supplementary Table 2.

ORDER	BINOMIAL NAME	COMMON NAME	POLYMORPHIC VARIATIONS
Artiodactyla	*Procapra gutturosa*	Mongolian gazelle	5OR N119 M140 S143 Y160 V172 N182 V221
	Bos taurus	Cow	6OR K115 M120 S154 C190 E218 Q234
	Cervus elaphus	Red deer	T98 P168 M208 E226
	Cervus elaphus canadensis	Elk	M132 T191 E226
	Odocoileus hemionus	Mule deer	A116 A136 Q171 N173 T177 M208 Q226 Q230
	Alces alces	Moose	S100 K109 M209 Q226
	Odocoileus virginianus	White-tailed deer	Q95 G96 S100 N103 A123 R151 V192 Q215 S225 Q226
	Rangifer tarandus	Reindeer	G96 A123 G129 S138 Y153 V169 N176 K207 R211 S225 Q226
	Ovis aries	Sheep	5OR Q101 M112 G127 A136 M137 S138 L141 I142 H143 N146 R151 Y152 R154 R167 P168 Q171 Y172 Q175 N176 H180 Q189 T195 T196 R211
	Capra hircus	Goat	Q101 W102 S106 T110 M112 G127 L133 M137 R139 L141 I142 H143 G145 N146 E151 R154 R159 P168 Q171 I185 T193 T194 F201 I208 R211 Q215 I218 T219 Q220 Q222
Carnivora	*Canis lupus familiaris*	Dog	S101 E163
Chiroptera	*Phyllostomus discolor*	Pale spear-nosed bat	S160
Perissodactyla	*Equus caballus ferus*	Horse	G68 W101 M111 S134 Y165 N175 N183 S233
	Equus caballus przewalskii	Przewalski's horse	W101 M111 Y130 Y165 N175 S233
Primates	*Saimiri sciureus*	Squirrel monkey	6OR K164
