# Supplementary figures and images for: A comprehensive phylogeny of mammalian PRNP gene reveals no influence of prion misfolding propensity on the evolution of this gene
Source: PLoS Pathog. 2025 Jun 25;21(6):e1013257. doi: 10.1371/journal.ppat.1013257 (PMC12208436; doi:10.1371/journal.ppat.1013257)

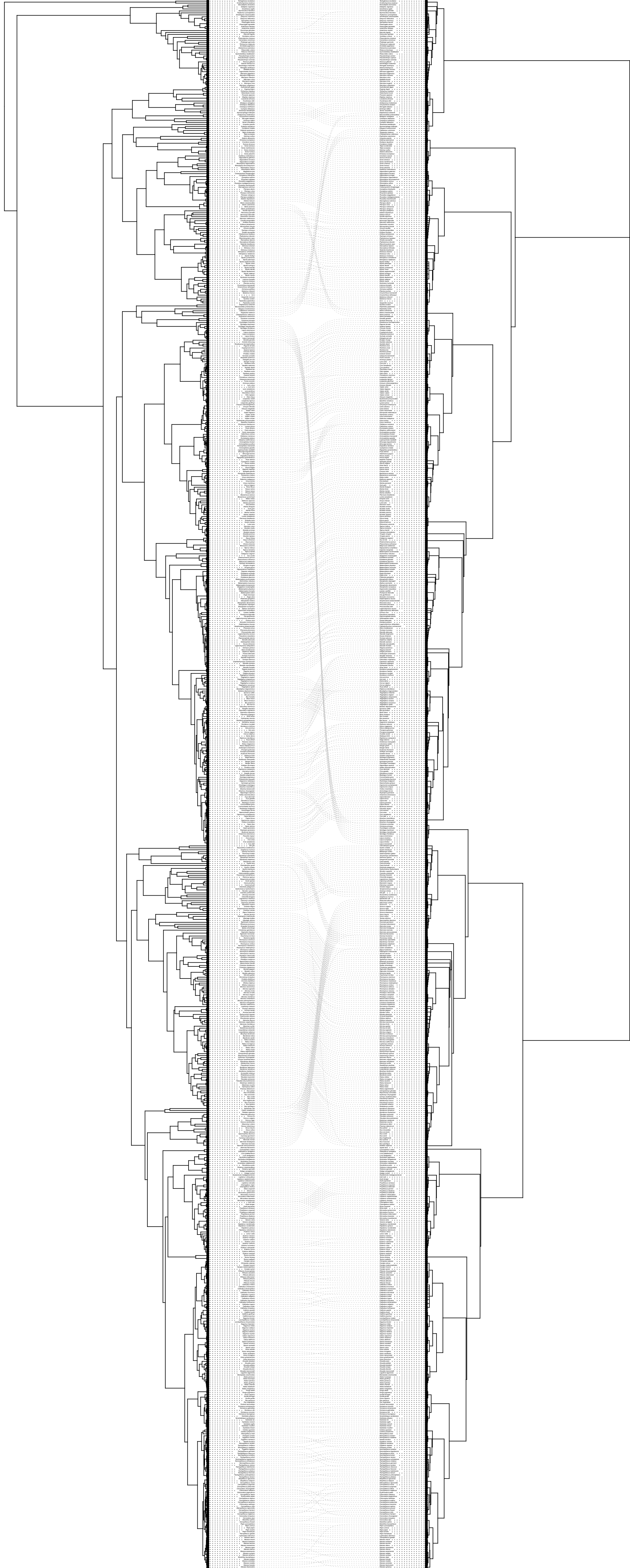

Supplement: S2 Fig — A tanglegram showing the topological differences between the PRNP gene-based tree and the species consensus tree (generated ad hoc using VertLife [49]. Lines connect the same species across both trees, and crossing lines indicate disagreement in tree structure. The Robinson-Foulds (RF) distance [50] between the two trees is 0.52, indicating that approximately 52% of the splits (bipartitions) differ. This suggests moderate topological incongruence between the gene-specific phylogeny and the overall species phylogeny. (PDF) [file ppat.1013257.s006.pdf]

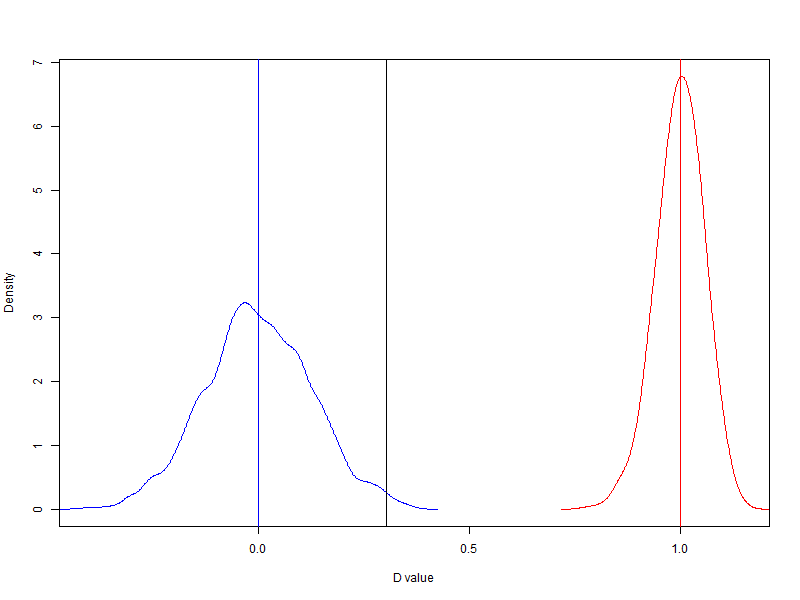

Supplement: S4 Fig — Density plots showing the distribution of the D statistic [65] under two null models: Brownian motion (blue) and random evolution (red), based on 1,000 permutations each. The observed D value for the PRNP misfolding trait is shown as a vertical dashed black line (D = 0.304). The observed value falls closer to the Brownian distribution, suggesting that the trait shows a phylogenetic signal consistent with gradual, tree-structured evolution rather than random distribution. Permutation tests yield p < 0.01 when compared to both null models. (PNG) [file ppat.1013257.s008.png]
