# Supplementary material for: A comprehensive phylogeny of mammalian PRNP gene reveals no influence of prion misfolding propensity on the evolution of this gene
Source: PLoS Pathog. 2025 Jun 25;21(6):e1013257. doi: 10.1371/journal.ppat.1013257 (PMC12208436; doi:10.1371/journal.ppat.1013257)
Supplement: S4 Table — The sequences, identified by their common species names as listed in S2 Table, that are part of each consensus are presented in this table next to the name assigned to each consensus and their corresponding order. (PDF) [file ppat.1013257.s009.pdf]

Supplementary Table 4. List of consensus sequences used in the phylogeentic study and of the species considered to build each consensus sequence. The sequences, identified by their common names as listed in Supplementary Table 2, that are part of each consensus are presented in this table next to the name of the consensus and their corresponding order.

Order	Consensus	Bootstrap (%)	Sequences included
Lagomorpha	Family Leporidae*	47	Sylvilagus floridanus, Sylvilagus transitionalis, Sylvilagus bachmani, Lepus timidus, Lepus americanus, Lepus oiostolus, Lepus europeaus, Lepus townsendii*
Chiroptera	Family Phyllostomidae	16	Desmodus rotundus, Phyllostomus hastatus, Phyllostomus discolor, Phyllostomus discolor 6OR N160, Phyllostomus discolor Δ38-47 N160, Tonatia saurophila, Trachops cirrhosus, Carollia perspicillata, Glossophaga soricina mutica, Anoura caudifer
	Genus Eptesicus	28	Eptesicus fuscus, Eptesicus nilssonii
	Species Pippistrellus pipistrellus	46	Pippistrellus pipistrellus 4OR, Pippistrellus pipistrellus 5OR
	Genus Myotis 1	46	Myotis planiceps, Myotis septentrionalis, Myotis findleyi, Myotis yumanensis
	Genus Myotis 2	21	Myotis daubentonii, Myotis myotis, Myotis nattereri, Myotis ricketti
	Genus Molossus	43	Molossus rufus, Molossus alvarezi, Molossus molossus
	Genus Pteropus	11	Pteropus medius, Pteropus rufus, Pteropus vampyrus, Pteropus pselaphon, Pteropus rodricensis, Pteropus alecto
	Genus Hipposideros	12	Hipposideros galeritus, Hipposideros larvatus, Hipposideros armiger, Hipposideros pendleburyi
	Genus Rhinolophus	12	Rhinolophus hipposideros, Rhinolophus affinis, Rhinolophus sinicus, Rhinolophus ferrumequinum
Artiodactyla	Family Cervidae	42	Alces alces andersonii M209, Alces alces andersonii I209, Alces alces, Alces alces Q109, Alces alces R100 I209, Alces alces I209, Capreolus pygargus, Capreolus capreolus, Odocoileus hemionus, Odocoileus hemionus F225, Odocoileus hemionus G116, Odocoileus hemionus L230, Odocoileus virginianus, Odocoileus virginianus H95, Odocoileus virginianus S96, Odocoileus virginianus I103, Odocoileus virginianus T123, Odocoileus virginianus S96 T123, Odocoileus virginianus S96 N100, Odocoileus virginianus N100 Odocoileus virginianus K226, Odocoileus virginianus R226, Odocoileus virginianus T103, Odocoileus virginianus clavium, Odocoileus virginianus V136 R171 S173 N177 I208, Odocoileus virginianus T103 H151, Odocoileus virginianus R96, Odocoileus virginianus H95 S96, Odocoileus virginianus K215, Rangifer tarandus, Rangifer tarandus C96 T123 D176, Rangifer tarandus Y225, Rangifer tarandus S129, Rangifer tarandus S129 M169, Rangifer tarandus N138, Rangifer tarandus D176, Rangifer tarandus F153 Q226, Rangifer tarandus caribou, Rangifer tarandus M207, Rangifer tarandus Q211, Cervus elaphus canadensis L132, Cervus elaphus I208, Cervus elaphus hanglu, Cervus elaphus canadensis A191 , Cervus elaphus canadensis, Cervus elaphus A98 Q226, Cervus elaphus A98 S168 Q226, Cervus elaphus hispanicus Q226, Cervus nippon, Przewalskium albirostris, Cervus hanglu yarkandensis, Axis axis, Cervus nippon, Cervus nippon, Dama dama, Elaphurus davidianus S138 I208 E226, Elaphurus davidianus N138 M208 Q226, Muntiacus crinifrons, Muntiacus muntjak, Muntiacus gongshanensis, Muntiacus reevesi, Rucervus eldii thamin, Hydropotes inermis 4OR Q226, Hydropotes inermis 5OR G170, Hydropotes inermis 5OR D96, Hydropotes inermis 5OR N100
	Family Bovidae	46	Aepyceros melampus, Beatragus hunteri, Damaliscus lunatus, Connochaetes gnou, Connochaetes taurinus, Antilope cervicapra, Nanger granti, Antidorcas marsupialis, Nanger dama, Nanger dama ruficollis, Saiga tatarica, Madoqua kirkii, Gazella dorcas, Gazella subgutturosa, Neotragus moschatus, Neotragus pygmaeus, Ourebia ourebi, Litocranius walleri, Procapra gutturosa, Procapra gutturosa 6OR S119 G143 H160, Procapra gutturosa 6OR S119 G143 H160 A172, Procapra gutturosa 6OR S182 A221, Procapra przewalskii 6OR, Procapra gutturosa 6OR S119 I140 G143 H160 A172, Eudorcas thomsonii, Oreotragus oreotragus, Connochaetes gnou, Connochaetes taurinus, Bison bison, Bubalus bubalis, Bubalus bubalis, Bubalus depressicornis Q204, Bubalus depressicornis R204, Bison bonasus, Bubalus bubalis 6OR G108, Syncerus caffer, Boselaphus tragocamelus, Tragelaphus oryx, Tragelaphus imberbis, Tragelaphus strepsiceros, Bos grunniens, Bos grunniens, Bos primigenius 6OR T106 G108 S154, Bos javanicus, Bos taurus, Bos taurus I120, Bos taurus K218, Bos taurus 7OR, Bos taurus 5OR, Bos taurus R115 R190, Bos javanicus, Bos taurus R234, Bos indicus, Bos frontalis, Bos mutus, Bos primigenius S106 S108 N154, Bos gaurus, Bos taurus, Bos taurus N154, Ammotragus lervia, Budorcas taxicolor, Capra hircus, Capra ibex, Capra pyrenaica, Capra sibirica, Capra falconeri, Capra hircus R101, Capra hircus G102, Capra hircus P110, Capra hircus T112, Capra hircus S127, Capra hircus Q133, Capra hircus I137, Capra hircus S139, Capra hircus F141, Capra hircus M142, Capra hircus T142, Capra hircus R143, Capra hircus D145, Capra hircus D146, Capra hircus S146, Capra hircus H151, Capra hircus H154, Capra hircus Q168, Capra hircus R106 R171, Capra hircus F185, Capra hircus P194, Capra hircus L201, Capra hircus T208, Capra hircus Q211, Capra hircus G211, Capra hircus R215, Capra hircus L218, Capra hircus I219, Capra hircus H220, Capra hircus K222, Capra hircus A127 S146, Capra hircus S146 H159, Capra hircus H159, Capra hircus S146 I193, Capra hircus I193, Capricornis sumatraensis, Hemitragus jayakari, Hemitragus hylocrius, Naemorhedus griseus, Oreamnos americanus, Ovibos moschatus, Capricornis crispus, Ovis aries, Ovis nivicola lydekkeri, Ovis vignei, Ovis dalli, Ovis aries, Ovis nivicola lydekkeri, Ovis orientalis musimon, Ovis canadensis, Ovis canadensis G138 R154, Ovis canadensis R154 G210, Ovis ammon, Ovis aries R171, Ovis aries V136, Ovis aries K176, Ovis aries T137, Ovis aries K171, Ovis aries I112, Ovis aries 6OR, Ovis aries V127, Ovis aries R101, Ovis aries A127 H154, Ovis aries T136, Ovis aries N138 L189, Ovis aries R138, Ovis aries K142, Ovis aries S146 L189, Ovis aries C151, Ovis aries F152, Ovis aries S167, Ovis aries L168, Ovis aries D172, Ovis aries E175, Ovis aries Y180, Ovis aries H154 L189, Ovis aries H154 R171 R189, Ovis aries S195, Ovis aries S196, Ovis aries G151, Ovis aries L189, Ovis aries Q114, Ovis aries E116, Ovis aries P118, Ovis aries V137, Ovis aries T138, Ovis aries S145, Ovis aries K149, Ovis aries K167, Ovis aries G169, Ovis aries H171, Ovis aries N171, Ovis aries F192, Ovis aries M142, Ovis aries H154, Ovis aries T112, Ovis aries S127, Ovis aries F141, Ovis aries R143, Pantholops hodgsonii, Rupicapra pyrenaica 4OR, Rupicapra pyrenaica 5OR, Addax nasomaculatus, Oryx dammah, Oryx gazella, Hippotragus equinus, Hippotragus niger niger, Hippotragus niger, Kobus ellipsiprymnus, Redunca redunca, Kobus megaceros, Kobus leche leche, Philantomba maxwellii, Cephalophus harveyi, Sylvicapra grimmia, Tragelaphus buxtoni, Tragelaphus scriptus, Tragelaphus eurycerus, Tragelaphus angasii
	Genus Moschus	29	Moschus berezovskii, Moschus moschiferus, Moschus chrysogaster
	Family Giraffidae	43	Giraffa camelopardalis tippelskirchi K175, Giraffa camelopardalis tippelskirchi 6OR R175, Giraffa camelopardalis antiquorum, Okapia johnstoni, Giraffa reticulata
	Genus Lama	35	Lama glama chaku, Lama guanicoe cacsilensis, Lama glama
	Genus Vicugna	35	Vicugna pacos huacaya, Vicugna pacos, Vicugna vicugna, Vicugna vicugna mensalis
	Genus Camelus	14	Camelus ferus bactrianus, Camelus dromedarius, Camelus dromedarius E134, Camelus dromedarius G134
Cetacea	Balaenoptera Spp.	10	Balaenoptera bonaerensis, Balaenoptera acutorostrata, Balaenoptera physalus, Balaenoptera musculus
	Family Ziphiidae	16	Hyperoodon ampullatus, Ziphius cavirostris, Mesoplodon biden, Mesoplodon densirostris, Mesoplodon europaeus, Mesoplodon stejnegeri
	Family Physeteridae	16	Kogia sima, Kogia breviceps, Physeter catodon
	Family Delphinidae	48	Lagenorhynchus obliquidens, Tursiops truncatus A234 S235, Tursiops truncatus K184, Orcinus orca Y234 G235, Stenella coeruleoalba, Stenella longirostris, Stenella clymene, Orcinus orca A234 S235, Lagenorhynchus albirostris, Grampus griseus, Lagenorhynchus acutus, Sousa chinensis, Tursiops aduncus , Globicephala melas, Delphinus delphis, Peponocephala electra, Feresa attenuata, Steno bredanensis, Cephalorhynchus commersonii, Stenella attenuata, Stenella frontalis, Tursiops truncatus N184 Y234 G235, Pseudorca crassidens
	Family Iniidae	48	Inia geoffrensis, Pontoporia blainvillei, Pontoporia blainvillei 6OR, Lipotes vexillifer
	Family Phocoenidae	48	Neophocaena asiaeorientalis A234 S235, Phocoena sinus 3OR A234 S235, Phocoena sinus Y234 G235, Phocoenoides dalli, Phocoena phocoena, Neophocaena phocaenoides, Neophocaena asiaeorientalis Y234 G235
Perissodactyla	Family Rhinocerontidae	4	Diceros bicornis minor, Ceratotherium simum cottoni, Ceratotherium simum simum, Diceros bicornis, Rhinoceros unicornis, Dicerorhinus sumatrensis harrissoni, Dicerorhinus sumatrensis sumatrensis
	Genus Equus	47	Equus przewalskii, Equus asinus, Equus ferus caballus, Equus przewalskii C130, Equus ferus caballus K175, Equus asinus, Equus ferus caballus G134 C165, Equus ferus caballus V68 D183, Equus ferus caballus N233, Equus przewalskii I111, Equus ferus caballus R101, Equus quagga boehmi, Equus grevyi, Equus africanus, Equus burchellii, Equus zebra hartmannae, Equus kiang
	Genus Manis	47	Manis tricuspis, Manis crassicaudata, Manis pentadactyla, Manis javanica, Manis gigantea, Manis temminckii, Manis tetradactyla, Manis culionensis
Carnivora	Family Canidae**	37	Urocyon littoralis, Canis mesomelas, Vulpes velox, Nyctereutes procyonoides, Urocyon cinereoargenteus, Vulpes lagopus, Vulpes zerda, Vulpes vulpes, Vulpes corsac, Vulpes ferrilata, Otocyon megalotis, Canis latrans, Canis lupus familiaris, Canis lupus familiaris D163, Canis lupus familiaris G101 D163, Canis adustus, Canis lupus familiaris G101, Lycaon pictus, Canis lupus dingo
	Family Ursidae	45	Tremarctos ornatus, Ursus arctos, Ursus maritimus, Helarctos malayanus, Ursus thibetanus thibetanus, Ailuropoda melanoleuca, Ursus americanus, Ursus thibetanus japonicus
	Subfamily Lutrinae	42	Enhydra lutris kenyoni, Enhydra lutris nereis, Lutra lutra, Enhydra lutris, Pteronura brasiliensis, Lontra canadensis, Aonyx cinerea

Carnivora	Subfamily Mustelinae	42	Mustela lutreola, Mustela putorius I113 F179, Meles meles, Mustela nigripes, Mustela nivalis, Mustela erminea, Mustela putorius M113 L179, Neovison vison, Martes pennanti, Martes flavigula, Martes foina, Eira barbara, Gulo gulo, Gulo gulo luscus, Martes zibellina, Mellivora capensis, Taxidea taxus jeffersonii
	Family Phocidae 1	26	Monachus schauinslandi, Mirounga angustirostris,Mirounga leonina
	Family Phocidae 2	15	Pusa hispida saimensis, Phoca groenlandica, Pusa sibirica, Halichoerus grypus
	Family Phocidae 3	22	Phoca largha, Cystophora cristata, Phoca vitulina
	Family Otariidae	14	Eumetopias jubatus, Otaria byronia, Arctocephalus gazella, Zalophus californianus, Arctocephalus forsteri, Arctocephalus pusillus, Callorhinus ursinus, Arctocephalus townsendi, Callorhinus ursinus
	Subfamily Pantherinae	10	Panthera onca, Panthera pardus, Panthera leo, Neofelis nebulosa, Panthera uncia, Panthera tigris altaica, Panthera tigris sumatrae, Panthera tigris jacksoni, Neofelis diardi
	Subfamily Felinae	37	Leopardus geoffroyi, Leopardus wiedii, Leopardus tigrinus, Lynx canadensis, Lynx lynx, Lynx rufus, Lynx pardinus, Felis silvestris catus, Felis chaus, Felis nigripes, Otocolobus manul, Puma yagouaroundi, Catopuma temminckii, Acinonyx jubatus
Sirenia	Order Sirenia	23	Dugong dugon, Hydrodamalis gigas, Trichechus manatus latirostris, Trichechus manatus
Hyracoidea	Order Hyracoidea	23	Heterohyrax brucei, Procavia capensis
Primates	Family Indriidae	14	Indri indri, Propithecus deckenii coronatus, Propithecus diadema, Propithecus edwardsi, Propithecus perrieri, Propithecus verreauxi, Propithecus coquereli, Propithecus tattersalli, Avahi laniger, Avahi peyrierasi
	Family Cheirogaleidae	14	Cheirogaleus medius, Cheirogaleus major, Microcebus griseorufus, Microcebus mittermeieri, Microcebus ravelobensis, Microcebus tavaratra, Microcebus murinus, Mirza coquereli, Mirza zaza
	Family Lemuridae	36	Prolemur simus, Lemur catta, Eulemur coronatus, Eulemur rufus, Eulemur sanfordi, Hapalemur griseus, Hapalemur alaotrensis, Hapalemur meridionalis, Hapalemur occidentalis, Eulemur rubriventer, Eulemur albifrons, Eulemur flavifrons, Eulemur fulvus, Eulemur macaco, Eulemur mongoz, Eulemur collaris, Eulemur rufifrons, Varecia variegata, Varecia rubra
	Genus Hylobates	12	Hylobates moloch, Hylobates pileatus, Hylobates lar, Hylobates syndactylus, Hylobates klossii, Hylobates muelleri, Hylobates agilis
	Genus Nomascus	35	Nomascus annamensis, Nomascus gabriellae, Nomascus leucogenys, Nomascus siki, Nomascus concolor
	Genus Ateles	21-13	Ateles hybridus, Ateles fusciceps, Ateles marginatus, Ateles belzebuth, Ateles chamek, Ateles geoffroyi
	Genus Alouatta	43	Alouatta belzebul, Alouatta palliata, Alouatta discolor, Alouatta juara, Alouatta macconnelli, Alouatta caraya, Alouatta seniculus puruensis
	Family Cebidae	14	Saguinus bicolor, Saguinus inustus, Saguinus labiatus , Saguinus mystax, Saguinus imperator, Saguinus oedipus, Saguinus midas, Leontopithecus rosalia, Leontopithecus chrysomelas, Callithrix jacchus N96 R219, Saguinus fuscicollis, Saguinus nigricollis, Callithrix pygmaea, Callithrix humeralifer, Callithrix humilis, Callithrix geofroyi, Callithrix jacchus S96 K219, Callithrix argentata, Callithrix kuhlii, Callimico goeldii, Cebus apella L48, Cebus apella R48, Cebus capucinus, Cebus imitator, Cebus olivaceus, Cebus unicolor, Cebus albifrons, Saimiri boliviensis, Saimiri sciureus, Saimiri sciureus, Saimiri macrodon, Saimiri oerstedii, Saimiri ustus, Saimiri sciureus, Saimiri cassiquiarensis
	Genus Cacajao	34	Cacajao ayresi, Cacajao melanocephalus, Cacajao calvus, Cacajao hosomi
	Genus Pithecia	22	Pithecia albicans, Pithecia chrysocephala, Pithecia pithecia, Pithecia hirsuta, Pithecia mittermeieri, Pithecia pissinatti, Pithecia vanzolinii, Pithecia irrorata
	Genus Callicebus	23	Callicebus lucifer, Callicebus lugens, Callicebus torquatus, Callicebus brunneus, Callicebus caligatus, Callicebus moloch, Callicebus cupreus, Callicebus dubius, Callicebus bernhardi, Callicebus hoffmannsi, Callicebus grovesi, Callicebus cinerascens, Callicebus miltoni, Callicebus donacophilus
	Subfamily Cercopithecinae 1	0.2	Macaca sylvanus, Mandrillus leucophaeus 6OR, Mandrillus leucophaeus Δ117-141, Mandrillus leucophaeus 2OR, Mandrillus leucophaeus 3OR, Mandrillus leucophaeus 4OR, Macaca cyclopis, Macaca assamensis, Macaca silenus, Macaca thibetana thibetana, Macaca nigra, Macaca leonina, Macaca maura, Macaca nemestrina, Macaca siberu, Macaca tonkeana, Miopithecus talapoin, Miopithecus ogouensis, Macaca fascicularis, Mandrillus sphinx, Macaca arctoides, Macaca mulatta, Macaca nemestrina, Macaca fuscata, Macaca fasciculari, Macaca radiata
	Subfamily Cercopithecinae 2	0.2	Cercocebus atys, Cercocebus torquatus, Allenopithecus nigroviridis, Cercopithecus mona, Cercopithecus campbelli lowei, Cercopithecus pogonias, Cercopithecus petaurista, Cercopithecus neglectus, Cercopithecus mitis, Cercopithecus albogularis, Cercopithecus ascanius, Cercopithecus lhoesti, Cercopithecus preussi, Cercopithecus solatus, Cercopithecus aethiops, Cercopithecus dianae, Chlorocebus sabaeus, Cercopithecus cephus, Chlorocebus pygerythrus, Cercopithecus hamlyni, Cercopithecus roloway, Cercopithecus patas, Cercopithecus patas, Cercocebus chrysogaster, Cercocebus atys, Papio papio, Papio cynocephalus, Papio ursinus, Papio anubis, Papio hamadryas, Cercocebus aterrimus, Theropithecus gelada, Theropithecus gelada
	Subfamily Colobinae	41	Trachypithecus francoisi, Colobus angolensis palliatus, Colobus guereza, Trachypithecus laotum, Rhinopithecus bieti, Trachypithecus francoisi, Trachypithecus poliocephalus, Piliocolobus tephrosceles, Piliocolobus kirkii, Trachypithecus phayrei crepuscula, Trachypithecus pileatus, Piliocolobus badius, Piliocolobus gordonorum, Trachypithecus auratus, Rhinopithecus roxellana, Pygathrix nigripes, Rhinopithecus strykeri, Trachypithecus obscurus, Trachypithecus germaini, Trachypithecus cristatus, Trachypithecus hatinhensis, Trachypithecus vetulus, Pygathrix nemaeus, Pygathrix cinerea, Nasalis larvatus, Presbytis comata, Presbytis melalophos mitrata, Colobus polykomos, Colobus angolensis palliatus 2OR, Colobus angolensis palliatus 3OR, Semnopithecus entellus, Semnopithecus hypoleucos , Semnopithecus priam, Semnopithecus schistaceus, Trachypithecus johnii
Rodentia	Suborder Sciuromorpha	26	Spermophilus beecheyi, Spermophilus tridecemlineatus, Cynomys ludovicianus, Spermophilus parryii, Cynomys gunnisoni, Spermophilus dauricus, Marmota flaviventris, Marmota marmota marmota, Marmota vancouverensis, Marmota marmota, Marmota himalayana, Marmota monax, Tamias sibiricus, Xerus inauris, Xerus rutilus, Sciurus lis, Sciurus vulgaris, Petaurista alborufus, Sciurus carolinensis, Sciurus niger, Glaucomys volans, Sciurus stramineus, Aplodontia rufa, Muscardinus avellanarius, Glis glis, Graphiurus murinus
	Subfamily Murinae 1	29	Tokudaia muenninki, Tokudaia tokunoshimensis, Tokudaia osimensis, Apodemus mystacinus, Apodemus fulvipectus, Apodemus sylvaticus, Apodemus speciosus, Rhabdomys pumilio, Rhabdomys dilectus, Arvicanthis niloticus, Grammomys surdaster
	Subfamily Murinae 2	30	Bandicota indica, Rattus exulans, Bandicota savilei, Berylmys berdmorei, Rattus argentiventer, Rattus nitidus, Rattus rattus, Rattus losea, Rattus tanezumi, Rattus norvegicus, Leopoldamys edwardsi, Leopoldamys sabanus
	Subfamily Murinae 3	21	Rhynchomys soricoides, Chiropodomys gliroides, Hydromys chrysogaster, Uromys caudimaculatus
	Mus Spp.	43	Mus caroli, Mus fragilicauda, Mus spicilegus, Mus spretus
	Subfamily Arvicolinae	4	Ellobius lutescens, Ellobius talpinus, Microtus oeconomus, Neodon shergylaensis, Microtus arvalis, Microtus fortis, Microtus agrestis, Microtus oregoni, Microtus californicus, Microtus pennsylvanicus, Microtus montanus, Microtus ochrogaster, Microtus richardsoni, Microtus richardsoni arvicoloides, Arvicola amphibius, Dicrostonyx torquatus
	Myodes Spp.	49	Myodes rutilus, Myodes gapperi
	Genus Peromyscus	12	Peromyscus aztecus, Peromyscus californicus insignis, Peromyscus eremicus, Peromyscus melanophrys, Peromyscus nudipes, Peromyscus leucopus, Peromyscus maniculatus bairdii, Peromyscus polionotus subgriseus, Peromyscus maniculatus sonoriensis, Peromyscus crinitus, Peromyscus nasutus, Peromyscus attwateri
	Family Spalacidae	15	Eospalax fontanierii, Rhizomys pruinosus, Spalax galili, Spalax ehrenbergi
	Family Dipodidae	15	Dipus sagitta, Jaculus jaculus, Salpingotus crassicauda, Allactaga sibirica, Allactaga bullata, Zapus hudsonius
	Family Heteromyidae	36	Dipodomys ordii, Dipodomys spectabilis, Dipodomys stephensi, Dipodomys merriami, Dipodomys merriami, Perognathus longimembris pacificus
	Infraorder Hystricognathi***	37	Heterocephalus glaber, Bathyergus suillus, Cryptomys darlingi, Cryptomys damarensis, Thryonomys swinderianus, Petromus typicus, Ctenomys sociabilis, Capromys pilorides, Myocastor coypus, Octodon degus, Octomys mimax, Tympanoctomys barrerae, Dasyprocta punctata, Coendou prehensilis, Erethizon dorsatum, Cavia porcellus, Cavia tschudii, Cavia aperea, Hydrochoerus hydrochaeris, Kerodon rupestris, Dolichotis patagonum, Chinchilla lanigera, Dinomys branickii, Lagostomus maximus

* O. cuniculus also belongs to this family but is clearly differentiated by bootstrap values

**Speothon and chrysocyon belong to this family but are separated by bootstrap values

*** Does not include all representatives from this infraorder included in this study
