# Supplementary material for: A comprehensive phylogeny of mammalian PRNP gene reveals no influence of prion misfolding propensity on the evolution of this gene
Source: PLoS Pathog. 2025 Jun 25;21(6):e1013257. doi: 10.1371/journal.ppat.1013257 (PMC12208436; doi:10.1371/journal.ppat.1013257)
Supplement: S1 Fig — 1146 different nucleotide sequences from 901 different mammalian species were used to perform the analysis as described. Values shown in each branch represent the bootstrap values obtained after 10,000,000 iterations. (PDF) [file ppat.1013257.s005.pdf]

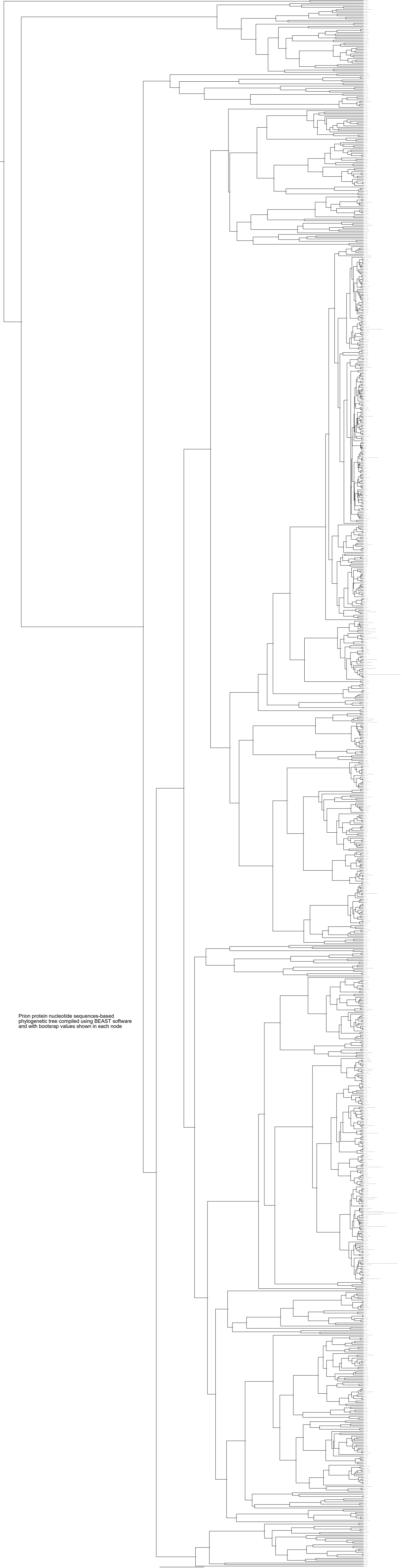

Prion protein nucleotide sequences-based phylogenetic tree compiled using BEAST software and with bootsrap values shown in each node
