# Supplementary material for: A comprehensive phylogeny of mammalian PRNP gene reveals no influence of prion misfolding propensity on the evolution of this gene
Source: PLoS Pathog. 2025 Jun 25;21(6):e1013257. doi: 10.1371/journal.ppat.1013257 (PMC12208436; doi:10.1371/journal.ppat.1013257)
Supplement: S3 Fig — The misfolding results have been mapped onto the PRNP phylogenetic tree. Stochastic character mapping was used to reconstruct ancestral states and plot them on the nodes of the tree as pies. Blue represents misfolding positive species whereas yellow represent species that have been unable to misfold. The pies fraction of the pie coloured in one or the other represents the fraction of the terminal nodes in which misfolding has been detected or not by PMSA. There is no grouping by prion disease resistant or susceptible amino acids. (PDF) [file ppat.1013257.s007.pdf]

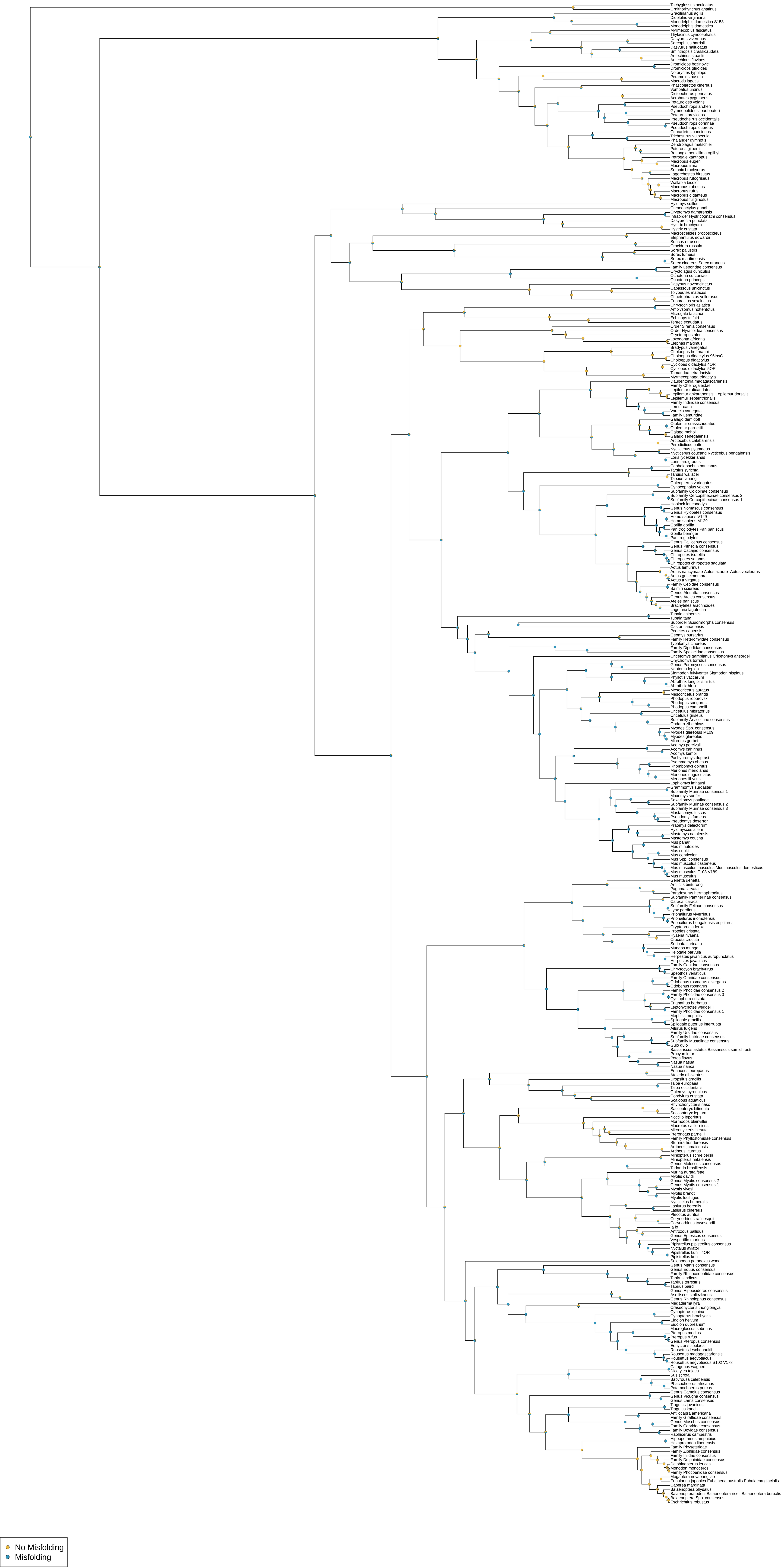
Tachyglossus aculeatus
Ornithorhynchus anatinus
Gracilinanus agilis
Didelphis virginiana
Monodelphis domestica S153
Monodelphis domestica
Myrmecobius fasciatus
Thylacinus cynocephalus
Dasyurus viverrinus
Sarcophilus harrisii
Dasyurus hallucatus
Sminthopsis crassicaudata
Antechinus stuartii
Antechinus flavipes
Dromiciops bozinovici
Dromiciops gliroides
Notoryctes typhlops
Perameles nasuta
Macrotis lagotis
Phascolarctos cinereus
Vombatus ursinus
Distoechurus pennatus
Acrobates pygmaeus
Petauroides volans
Pseudochirops archeri
Gymnobelideus leadbeateri
Petaurus breviceps
Pseudocheirus occidentalis
Pseudochirops corinnae
Pseudochirops cupreus
Cercartetus concinnus
Trichosurus vulpecula
Phalanger gymnotis
Dendrolagus matschiei
Potorous gilbertii
Bettongia penicillata ogilbyi
Petrogale xanthopus
Macropus eugenii
Macropus irma
Setonix brachyurus
Lagorchestes hirsutus
Macropus rufogriseus
Wallabia bicolor
Macropus robustus
Macropus rufus
Macropus giganteus
Macropus fuliginosus
Hylomys suillus
Ctenodactylus gundi
Cryptomys damarensis
Infraorder Hystricognathi consensus
Dasyprocta punctata
Hystrix brachyura
Hystrix cristata
Macroscelides proboscideus
Elephantulus edwardii
Suncus etruscus
Crocidura russula
Sorex palustris
Sorex fumeus
Sorex maritimensis
Sorex cinereus Sorex araneus
Family Leporidae consensus
Oryctolagus cuniculus
Ochotona curzoniae
Ochotona princeps
Dasypus novemcinctus
Cabassous unicinctus
Tolypeutes matacus
Chaetophractus vellerosus
Euphractus sexcinctus
Chrysochloris asiatica
Amblysomus hottentotus
Microgale talazaci
Echinops telfairi
Tenrec ecaudatus
Order Sirenia consensus
Order Hyracoidea consensus
Orycteropus afer
Loxodonta africana
Elephas maximus
Bradypus variegatus
Choloepus hoffmanni
Choloepus didactylus 96InsG
Choloepus didactylus
Cyclopes didactylus 4OR
Cyclopes didactylus 5OR
Tamandua tetradactyla
Myrmecophaga tridactyla
Daubentonia madagascariensis
Family Cheirogaleidae
Lepilemur ruficaudatus
Lepilemur ankaranensis Lepilemur dorsalis
Lepilemur septentrionalis
Family Indriidae consensus
Lemur catta
Varecia variegata
Family Lemuridae
Galago demidoff
Otolemur crassicaudatus
Otolemur garnettii
Galago moholi
Galago senegalensis
Arctocebus calabarensis
Perodicticus potto
Nycticebus pygmaeus
Nycticebus coucang Nycticebus bengalensis
Loris lydekkerianus
Loris tardigradus
Cephalopachus bancanus
Tarsius syrichta
Tarsius wallacei
Tarsius lariang
Galeopterus variegatus
Cynocephalus volans
Subfamily Colobinae consensus
Subfamily Cercopithecinae consensus 2
Subfamily Cercopithecinae consensus 1
Hoolock leuconedys
Genus Nomascus consensus
Genus Hylobates consensus
Homo sapiens V129
Homo sapiens M129
Gorilla gorilla
Pan troglodytes Pan paniscus
Gorilla beringei
Pan troglodytes
Genus Callicebus consensus
Genus Pithecia consensus
Genus Cacajao consensus
Chiropotes israelita
Chiropotes satanas
Chiropotes chiropotes sagulata
Aotus lemurinus
Aotus nancymaae Aotus azarae Aotus vociferans
Aotus griseimembra
Aotus trivirgatus
Family Cebidae consensus
Saimiri sciureus
Genus Alouatta consensus
Genus Ateles consensus
Ateles paniscus
Brachyteles arachnoides
Lagothrix lagotricha
Tupaia chinensis
Tupaia tana
Suborder Sciuromorpha consensus
Castor canadensis
Pedetes capensis
Geomys bursarius
Family Heteromyidae consensus
Typhlomys cinereus
Family Dipodidae consensus
Family Spalacidae consensus
Cricetomys gambianus Cricetomys ansorgei
Onychomys torridus
Genus Peromyscus consensus
Neotoma lepida
Sigmodon fulviventer Sigmodon hispidus
Phyllotis vaccarum
Abrothrix longipilis hirtus
Abrothrix hirta
Mesocricetus auratus
Mesocricetus brandti
Phodopus roborovskii
Phodopus sungorus
Phodopus campbelli
Cricetulus migratorius
Cricetulus griseus
Subfamily Arvicolinae consensus
Ondatra zibethicus
Myodes Spp. consensus
Myodes glareolus M109
Myodes glareolus
Microtus gerbei
Acomys percivali
Acomys cahirinus
Acomys kempi
Pachyuromys duprasi
Psammomys obesus
Rhombomys opimus
Meriones meridianus
Meriones unguiculatus
Meriones libycus
Lophiomys imhausi
Grammomys surdaster
Subfamily Murinae consensus 1
Maxomys surifer
Saxatilomys paulinae
Subfamily Murinae consensus 2
Subfamily Murinae consensus 3
Mastacomys fuscus
Pseudomys fumeus
Pseudomys desertor
Praomys delectorum
Hylomyscus alleni
Mastomys natalensis
Mastomys coucha
Mus pahari
Mus minutoides
Mus cookii
Mus cervicolor
Mus Spp. consensus
Mus musculus castaneus
Mus musculus musculus Mus musculus domesticus
Mus musculus F108 V189
Mus musculus
Genetta genetta
Arctictis binturong
Paguma larvata
Paradoxurus hermaphroditus
Subfamily Pantherinae consensus
Caracal caracal
Subfamily Felinae consensus
Lynx pardinus
Prionailurus viverrinus
Prionailurus iriomotensis
Prionailurus bengalensis euptilurus
Cryptoprocta ferox
Proteles cristata
Hyaena hyaena
Crocuta crocuta
Suricata suricatta
Mungos mungo
Helogale parvula
Herpestes javanicus auropunctatus
Herpestes javanicus
Family Canidae consensus
Chrysocyon brachyurus
Speothos venaticus
Family Otariidae consensus
Odobenus rosmarus divergens
Odobenus rosmarus
Family Phocidae consensus 2
Family Phocidae consensus 3
Cystophora cristata
Erignathus barbatus
Leptonychotes weddellii
Family Phocidae consensus 1
Mephitis mephitis
Spilogale gracilis
Spilogale putorius interrupta
Ailurus fulgens
Family Ursidae consensus
Subfamily Lutrinae consensus
Subfamily Mustelinae consensus
Gulo gulo
Bassariscus astutus Bassariscus sumichrasti
Procyon lotor
Potos flavus
Nasua nasua
Nasua narica
Erinaceus europaeus
Atelerix albiventris
Uropsilus gracilis
Talpa europaea
Talpa occidentalis
Galemys pyrenaicus
Condylura cristata
Scalopus aquaticus
Rhynchonycteris naso
Saccopteryx bilineata
Saccopteryx leptura
Noctilio leporinus
Mormoops blainvillei
Macrotus californicus
Micronycteris hirsuta
Pteronotus parnellii
Family Phyllostomidae consensus
Sturnira hondurensis
Artibeus jamaicensis
Artibeus lituratus
Miniopterus schreibersii
Miniopterus natalensis
Genus Molossus consensus
Tadarida brasiliensis
Murina aurata feae
Myotis davidii
Genus Myotis consensus 2
Genus Myotis consensus 1
Myotis vivesi
Myotis brandtii
Myotis lucifugus
Nycticeius humeralis
Lasiurus borealis
Lasiurus cinereus
Plecotus auritus
Corynorhinus rafinesquii
Corynorhinus townsendii
Ia io
Antrozous pallidus
Genus Eptesicus consensus
Vespertilio murinus
Pipistrellus pipistrellus consensus
Nyctalus aviator
Pipistrellus kuhlii 4OR
Pipistrellus kuhlii
Solenodon paradoxus woodi
Genus Manis consensus
Genus Equus consensus
Family Rhinocerotidae consensus
Tapirus indicus
Tapirus terrestris
Tapirus bairdii
Genus Hipposideros consensus
Aselliscus stoliczkanus
Genus Rhinolophus consensus
Megaderma lyra
Craseonycteris thonglongyai
Cynopterus sphinx
Cynopterus brachyotis
Eidolon helvum
Eidolon dupreanum
Macroglossus sobrinus
Pteropus medius
Pteropus rufus
Genus Pteropus consensus
Eonycteris spelaea
Rousettus leschenaultii
Rousettus madagascariensis
Rousettus aegyptiacus
Rousettus aegyptiacus S102 V178
Catagonus wagneri
Dicotyles tajacu
Sus scrofa
Babyrousa celebensis
Phacochoerus africanus
Potamochoerus porcus
Genus Camelus consensus
Genus Vicugna consensus
Genus Lama consensus
Tragulus javanicus
Tragulus kanchil
Antilocapra americana
Family Giraffidae consensus
Genus Moschus consensus
Family Cervidae consensus
Family Bovidae consensus
Raphicerus campestris
Hippopotamus amphibius
Hexaprotodon liberiensis
Family Physeteridae
Family Ziphiidae consensus
Family Iniidae consensus
Family Delphinidae consensus
Delphinapterus leucas
Monodon monoceros
Family Phocoenidae consensus
Megaptera novaeangliae
Eubalaena japonica Eubalaena australis Eubalaena glacialis
Caperea marginata
Balaenoptera physalus
Balaenoptera edeni Balaenoptera ricei Balaenoptera borealis
Balaenoptera Spp. consensus
Eschrichtius robustus
No Misfolding
Misfolding
